# Unravelling the different components of nonphotochemical quenching using a novel analytical pipeline

**DOI:** 10.1111/nph.20271

**Published:** 2024-11-15

**Authors:** Lennart A. I. Ramakers, Jeremy Harbinson, Emilie Wientjes, Herbert van Amerongen

**Affiliations:** ^1^ Laboratory of Biophysics Wageningen University 6708WE Wageningen the Netherlands; ^2^ MicroSpectroscopy Research Facility Wageningen University 6708WE Wageningen the Netherlands

**Keywords:** *Arabidopsis thaliana*, multivariate analysis, nonphotochemical quenching, PsbS, xanthophyll cycle, zeaxanthin

## Abstract

- Photoprotection in plants includes processes collectively known as nonphotochemical quenching (NPQ), which quench excess excitation‐energy in photosystem II. NPQ is triggered by acidification of the thylakoid lumen, which leads to PsbS‐protein protonation and violaxanthin de‐epoxidase activation, resulting in zeaxanthin accumulation. Despite extensive study, questions persist about the mechanisms of NPQ.
- We have set up a novel analytical pipeline to disentangle NPQ induction curves measured at many light intensities into a limited number of different kinetic components. To validate the method, we applied it to Chl‐fluorescence measurements, which utilised the saturating‐pulse methodology, on wild‐type (wt) and zeaxanthin‐lacking (*npq1*) *Arabidopsis thaliana* plants. NPQ induction curves in wt and *npq1* can be explained by four components (α, β, γ and δ).
- The fastest two (β and γ) correlate with pH difference formed across the thylakoid membrane in wt and *npq1*. In wt, the slower component (α) appears to be due to the formation of zeaxanthin‐related quenching whilst for *npq1*, this component is ‘replaced’ by a slower component (δ), which reflects a photoinhibition‐like process that appears in the absence of zeaxanthin‐induced quenching.
- Expanding this approach will allow the effects of mutations and other abiotic‐stress factors to be directly probed by changes in these underlying components.

## Introduction

Photosynthesis is arguably one of the most important biological processes in Nature (Blankenship, 2008; Johnson, 2016). In this process, the incoming solar radiation is captured by photosynthetic organisms and converted to chemical energy, and so underpins most of the food chains on Earth (Nelson & Yocum, 2006; Blankenship, 2008; Johnson, 2016). However, if oxygenic photosynthetic organisms are exposed to light intensities at which the rate of photon absorption exceeds the rate of photochemical quenching of excitations and thus photosynthetic metabolism, the excess excitations present within the photosystems can cause photodamage. Specifically, high light results in a large portion of the reaction centres (RCs) within the photosystems being in the closed state. With increasing irradiance, it is more likely that an excitation encounters a closed RC leading to triplet states being formed by back reactions within the reaction centre, which in turn can produce singlet oxygen (Vass, 2011; Telfer, 2014). In order to minimise such photodamage, oxygenic photosynthetic organisms activate photoprotective mechanisms, designed to safely remove excess excitations from the photosystems via nonphotochemical quenching (NPQ) (Horton *et al*., 1996; Demmig‐Adams & Adams, 1996b; De Bianchi *et al*., 2010; Ruban *et al*., 2012). In photosystem II (PSII), NPQ is mediated by several different molecular mechanisms that act together to quench excitations (Demmig‐Adams & Adams, 1996a,b; D'Haese *et al*., 2004; Li *et al*., 2004, 2009; Johnson *et al*., 2009; Jahns & Holzwarth, 2012; Ruban *et al*., 2012; Sylak‐Glassman *et al*., 2014; Goldschmidt‐Clermont & Bassi, 2015; Armbruster *et al*., 2016; Ruban, 2016, 2019; Farooq *et al*., 2018; Townsend *et al*., 2018; Van Amerongen & Chmeliov, 2020; Ruban & Wilson, 2021; Long *et al*., 2022). The fastest of these mechanisms are triggered by the accumulation of protons in the lumenal space, and these processes have been studied for several decades (Demmig‐Adams & Adams, 1996a,b; D'Haese *et al*., 2004; Li *et al*., 2004, 2009; Johnson *et al*., 2009; Jahns & Holzwarth, 2012; Ruban *et al*., 2012; Sylak‐Glassman *et al*., 2014; Goldschmidt‐Clermont & Bassi, 2015; Armbruster *et al*., 2016; Ruban, 2016, 2019; Townsend *et al*., 2018; Ruban & Wilson, 2021; Long *et al*., 2022). These processes are associated with the protonation of the PsbS protein and the activation of the violaxanthin de‐epoxidase enzyme (VDE) leading to the accumulation of zeaxanthin via antheraxanthin. Whilst these processes are known to be important for NPQ, many studies of the kinetics of their induction and relaxation reveal a significant latency in the overall NPQ response (Kromdijk *et al*., 2016; Ruban, 2017; Wang *et al*., 2020). This latency is thought to make photosynthesis significantly less efficient upon a decrease in the actinic light intensity, whilst upon a sudden increase in light intensity, this latency could temporarily leave the photosynthetic apparatus under protected. Accordingly, these processes have been widely studied with a view to improving the overall efficiency of photosynthesis and photoprotection (Kromdijk *et al*., 2016; De Souza *et al*., 2022).

Typically, the molecular processes involved in the NPQ response are probed *in vivo* utilising either changes in the steady‐state fluorescence and absorption spectra of photosynthetic organisms following exposure to, or changes in, actinic light. Several studies have correlated particular changes in the absorption spectra with the activation of VDE leading to the accumulation of zeaxanthin (Bilger & Björkman, 1990; Li *et al*., 2000; Johnson *et al*., 2009) and the formation of a quenching species thought to be associated with the protonation of PsbS (Johnson & Ruban, 2010). In addition to these changes, it has also been noted that the size of the *trans*‐thylakoid voltage can be probed by monitoring changes in the absorption spectra due to electrochromic shifts (ECS) originating from the electric field generated by the *trans*‐thylakoid voltage (Bailleul *et al*., 2010), and by monitoring changes in the far‐red part of the spectra the oxidation state of the PSI RC can be measured (Harbinson & Woodward, 1987; Klughammer & Schreiber, 1998). Such studies have been used to explore different aspects of NPQ and have yielded some insights into the induction of the underlying processes. However, these measurements are broadly speaking difficult to perform and analyse due to the complexity created by the high degree of convolution of a large number of different chromophore containing molecular species with nontrivial absorption spectra.

Alternatively, photosynthesis can also be monitored *in vivo* by changes in the Chl fluorescence yield (Harbinson, 2018). Whilst there are some experimental indications that there may also be a small degree of variable PSI fluorescence (Lazár, 2013; Schreiber & Klughammer, 2021; Schreiber, 2023), it is generally accepted to be minor in comparison with variable fluorescence from PSII. Therefore, changes in Chl fluorescence yield are normally only useful for examining PSII (Harbinson, 2018), even though fluorescence‐derived PSII parameters are often in error due to the presence of PSI fluorescence. Commonly, fluorescence yield is measured using pulse–amplitude–modulated (PAM) fluorometry (Maxwell & Johnson, 2000; Baker, 2008; Harbinson, 2018). Whilst using only Chl fluorescence decreases the overall number of distinct processes that can be monitored independently, the variables that can be extracted by applying the saturating pulse method (often used in PAM measurements) and utilising the Butler model (Butler, 1978) describe important photosynthetic parameters such as the proxies for the quantum yield of PSII (ϕ_PSII_), the fraction of open PSII RCs (qP) and NPQ (Maxwell & Johnson, 2000). Due to its sensitivity, this technique has been widely applied to study photosynthetic phenomena such as NPQ. In the case of NPQ, this parameter can be calculated directly from the PAM data using the Stern–Volmer equation ($\mathrm{NPQ}=\left(F_{m}-F_{m}^{\prime }\right)/F_{m}^{\prime }$, where $F_{m}$ is the maximum fluorescence yield with closed RCs in the dark adapted state and $F_{m}^{\prime }$ is the maximum fluorescence yield with closed RCs in the presence of NPQ) (Bilger & Björkman, 1990; Maxwell & Johnson, 2000; Harbinson, 2018). Over the past few decades, applying this technique to both wild‐type (wt) and mutant plants has illustrated that there are many processes contributing to the overall NPQ developed by vascular plants in response to the *trans*‐thylakoid membrane proton potential difference established during illumination and the associated decrease in lumen pH that ensues. These mechanisms include but are not limited to the protonation of the PsbS protein and possibly the antenna complexes (Li *et al*., 2004; Johnson & Ruban, 2010; Mou *et al*., 2013; Dong *et al*., 2015; Ruan *et al*., 2023), the activation of the xanthophyll cycle (Bilger & Björkman, 1990; Jahns & Holzwarth, 2012), and photoinhibition (qI) (Taylor *et al*., 2019; Vetoshkina *et al*., 2023). As multiple processes play a role on similar timescales, it has thus far been difficult, or even impossible, to unambiguously separate the contribution of the different mechanisms in time. One possible approach to disentangle these processes is the recently developed frequency domain analysis of Chl fluorescence yield. However, due to the complex nature of the data, these approaches are not yet capable to quantitatively discriminate between these different processes (Nedbal & Lazár, 2021; Niu *et al*., 2023, 2024).

Here, we develop a novel multivariate analysis pipeline to deconvolute a data set of NPQ induction curves, obtained via PAM fluorometry (using red actinic light, Supporting Information Fig. S1b, to minimise chloroplast movement (Baránková *et al*., 2016)), to reveal the induction profiles of different NPQ components. Focussing on data sets for wt and *npq1* (zeaxanthin‐lacking) *A. thaliana*, this pipeline reveals four distinct components (α, β, γ and δ). Comparison with chemical treatments and previous studies allows the molecular processes associated with these components to be assigned. This allows the contributions of each of the different mechanisms underlying NPQ to be unambiguously identified directly from Chl fluorescence yield measurements for the first time.

## Materials and Methods

### Materials

All *A. thaliana* ((L.) Heynh.) plants were grown in a Hettich ESP PRC 1200 WL growth cabinet (Hettich; https://www.hettichbenelux.com). The cabinet was set to grow plants in short‐day conditions (8 h : 16 h, light : dark) at an actinic light intensity of 125 μmol m^−2^ s^−1^ (spectrum shown in Fig. S1a), a day time temperature of 24°C, a night‐time temperature of 22°C and a relative humidity of 60%. Wild‐type and *npq1* seeds of *A. thaliana* (Columbia‐0) were sown on soil and allowed to germinate and grow into seedlings for *c*. 2 wk. After this initial growth period, the seedlings were transferred to separate pots and allowed to grow for a further 4–5 wk. All leaves were measured at a similar developmental stage of between 42 and 47 d.

Nigericin and 3‐(3,4‐dichlorophenyl)‐3,3‐dimethylurea (DCMU) were purchased from Sigma‐Aldrich. d,l‐dithiothreitol (DTT) was purchased from Fluka chemicals; all chemicals were used without any further purification steps. Stock solutions of 50 mM DTT, 5 mM nigericin and 5 mM DCMU were prepared using water for the DTT and ethanol for the nigericin and DCMU. These stocks were then used to prepare aqueous solutions of 5 mM DTT, 5 mM DTT and 50 μM nigericin and 50 μM DCMU. These solutions were prepared from their respective stock solutions using a 50‐ml volumetric flask. Aliquots of these solutions were used to infiltrate wt and *npq1 A. thaliana* leaves for 2 h before measurement.

### Pulse amplitude modulated (PAM) fluorometry

All experiments were carried out using a Waltz PAM‐101 Fluorimeter (Walz; https://www.walz.com) coupled to a red actinic light source controlled via a microcontroller (ESP32 – Espressif Systems; https://www.espressif.com, spectrum shown in Fig. S1b). Briefly, *A. thaliana* leaves, dark‐adapted overnight, were detached from the plant just before the measurement and placed into a small amount of water in a Petri dish. Using a multicore optical fibre, the measuring light and saturating pulses (0.8 s duration and an intensity of 7000 μmol m^−2^s^−1^) of the PAM‐101 were directed onto the adaxial side of the leaf, near the midpoint of the leaf (but not including the midvein). The emitted Chl fluorescence was also detected utilising this multicore optical fibre. The actinic light scheme and saturating pulse sequence were generated and controlled by a batch file in the associated WinControl‐3.30 Waltz Pam software. This batch file sent a trigger signal to the ESP32 microprocessor at the start of the experiment to initiate the actinic light scheme, which consisted of 25 s of darkness, followed by 10 min of illumination at a user‐defined intensity, ending with a further 5 min of post‐illumination dark recovery. For the saturating pulses, the following sequence was used. An initial saturating pulse was utilised to measure the dark‐adapted state of the leaf at the start of the experiment, this pulse was followed by a series of pulses spaced at 10‐s intervals for the first 2 min of illumination. Finally, a series of saturating pulses, spaced at 30‐s intervals, were used for the rest of the experiment (hereafter, this pulse sequence is referred to as the high‐resolution pulse sequence). For wt, additional measurements were performed using a simplified saturating pulse regime with each pulse being spaced at 60‐s intervals throughout the measurement (hereafter referred to as the low‐resolution pulse sequence). This low‐resolution sequence was used to explore any perturbations in NPQ generated by the more complicated high‐resolution sequence applied in the other measurements. All measurements were repeated at least five times. NPQ ($\mathrm{NPQ}=\left(F_{m}-F_{m}^{\prime }\right)/F_{m}^{\prime }$) and qP ($\mathrm{qP}=\left(F_{m}^{\prime }-F^{\prime }\right)/\left(F_{m}^{\prime }-F_{o}^{\prime }\right)$) were then calculated for each measurement to generate an NPQ induction curve data set for each of the plants explored.

### Multivariate analysis pipeline

All of the data analysis was performed utilising a custom‐made Python3.9 script. This script implements a multivariant pipeline consisting of principal component analysis (PCA), full harmonic phasor analysis (FH‐PhA; equations outlined in Notes S1.1) and non‐negative matrix factorisation (NMF; algorithm outlined in Notes S1.2; Fig. S2) to objectively deconvolute the NPQ induction curves into several distinct components with distinct induction kinetics. Briefly, the combination of PCA and FH‐PhA allows the identification of the minimum number of components with distinct induction kinetics underpinning the data set. The PCA extracts components that vary strongly as the actinic light intensity changes (with respect to the biological variance), whereas the PCA‐guided FH‐PhA identifies any additional components, which vary weakly as the actinic light intensity changes, again with respect to the biological variance. Together, these multivariate techniques yield approximate contributions of these components for each induction curve in the data set. These approximate contributions are then used as initial conditions in the NMF step, allowing the data set to be accurately deconvoluted into the components identified in the PCA and FH‐PhA steps. This pipeline and each of its steps is further outlined in the results section using the wt *A. thaliana* data set as an example.

## Results

### The novel multivariate NPQ analysis pipeline and the wt NPQ induction data set

**Fig. 1:**
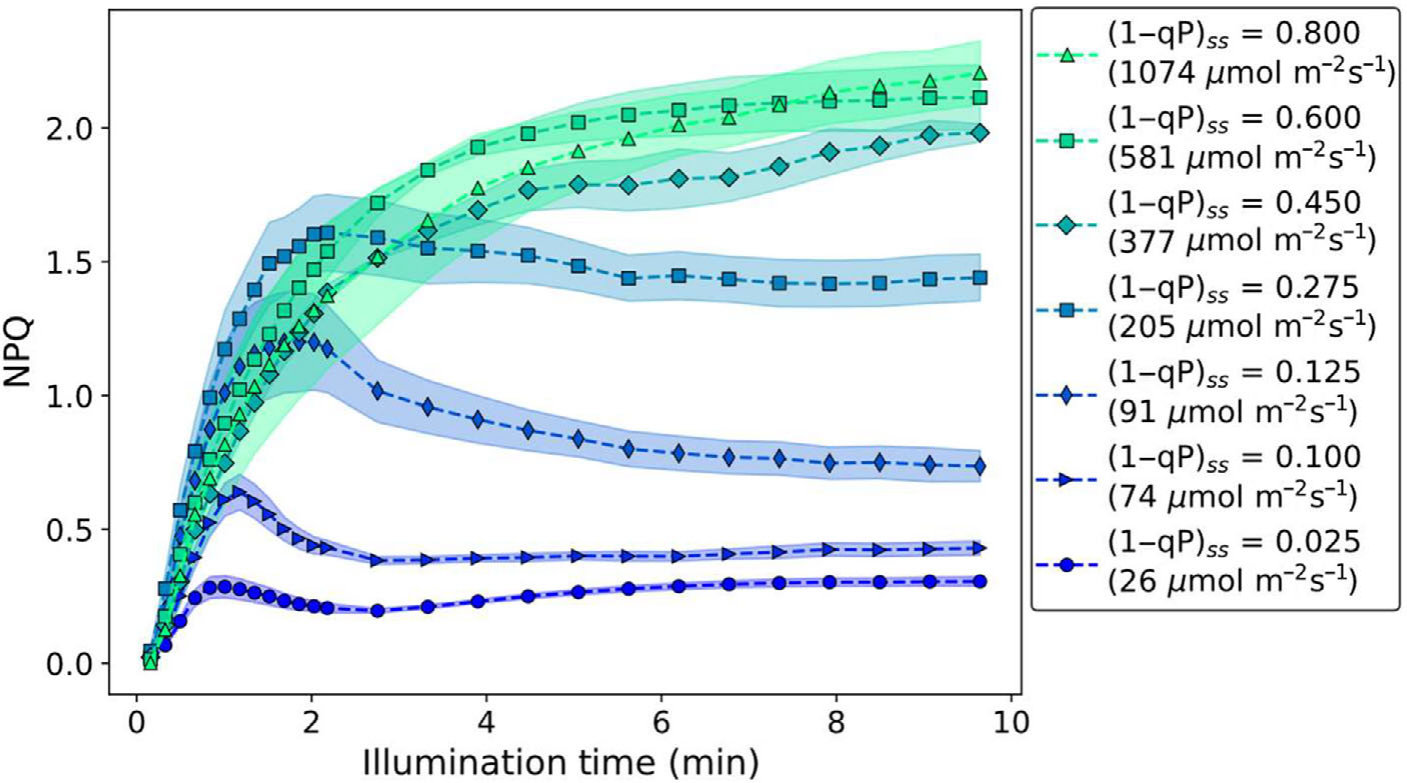
Overview of wild‐type *Arabidopsis thaliana* nonphotochemical quenching (NPQ) measured at different actinic light intensities. Wild‐type *A. thaliana* nonphotochemical quenching induction curves obtained (using the high‐resolution pulse sequence) at a range of different actinic light intensities, leading to differing values of (1−qP)_ss_. Each curve is the average of five individual measurements carried out on separate leaves, the shaded area shows the associated SE.

NPQ is commonly calculated utilising fluorescence yield parameters obtained from steady‐state fluorescence measurements via the Stern–Volmer equation. Whilst this is a remarkably simple equation, several different PSII‐associated NPQ processes are known to induce and relax with a range of differing kinetics. The presence of these processes is partially revealed by the changes in the NPQ induction curves measured over a range of different light intensities. As the actinic light intensity increases, a larger portion of PSII reaction centres was found to be closed at the end of the illumination period ((1−qP)_ss_, Fig. S3). In the following summary of our results, we will plot the induction curves of NPQ as a function of (1−qP)_ss_ instead of grouping them according to light intensity (Fig. S3). Average NPQ induction curves obtained at a range of different (1−qP)_ss_ values are shown in Fig. 1.

At low actinic light intensities (< 91 μmol m^−2^s^−1^), NPQ induction curves with intricate line shapes are observed. At these low light intensities, only a small portion of the RCs remains closed after 10 min of illumination ((1−qP)_ss_ < 0.125, Fig. 1). Specifically, under these light intensities, the NPQ induction curves have a local NPQ maximum, typically occurring within the first few minutes of illumination, followed by a decrease in a local minimum before gradually increasing again to a higher steady‐state NPQ value. Furthermore, as the actinic light intensity increases (from 26 to 205 μmol m^−2^s^−1^), the local NPQ maximum occurs at longer illumination times, increasing from *c*. 1 to 2 min of illumination. In contrast to this behaviour, at high light intensities (>> 205 μmol m^−2^s^−1^), the NPQ induction follows a simple monotonically increasing curve ((1−qP)_ss_ ≥ 0.45, Fig. 1). Between these extremes, the line shape of the NPQ induction curves exhibits a combination of these features. These characteristic line shapes and changes are also seen for the data set obtained using the low‐resolution pulse sequence (Fig. S4). However, these intermediate curves cannot be explained as a simple linear combination of these low light (26 μmol m^−2^s^−1^, blue circles, Fig. 1) and high light (1074 μmol m^−2^s^−1^, green triangles, Fig. 1) curves (Fig. S5a–e). This highlights the fact that there are intricate line shape changes, which occur as the actinic light intensity increases, with the largest discrepancy between this simple linear‐combination model and the induction curves occurring between (1−qP)_ss_ ~ 0.1 and (1−qP)_ss_ ~ 0.4 (Fig. S5f).

**Fig. 2:**
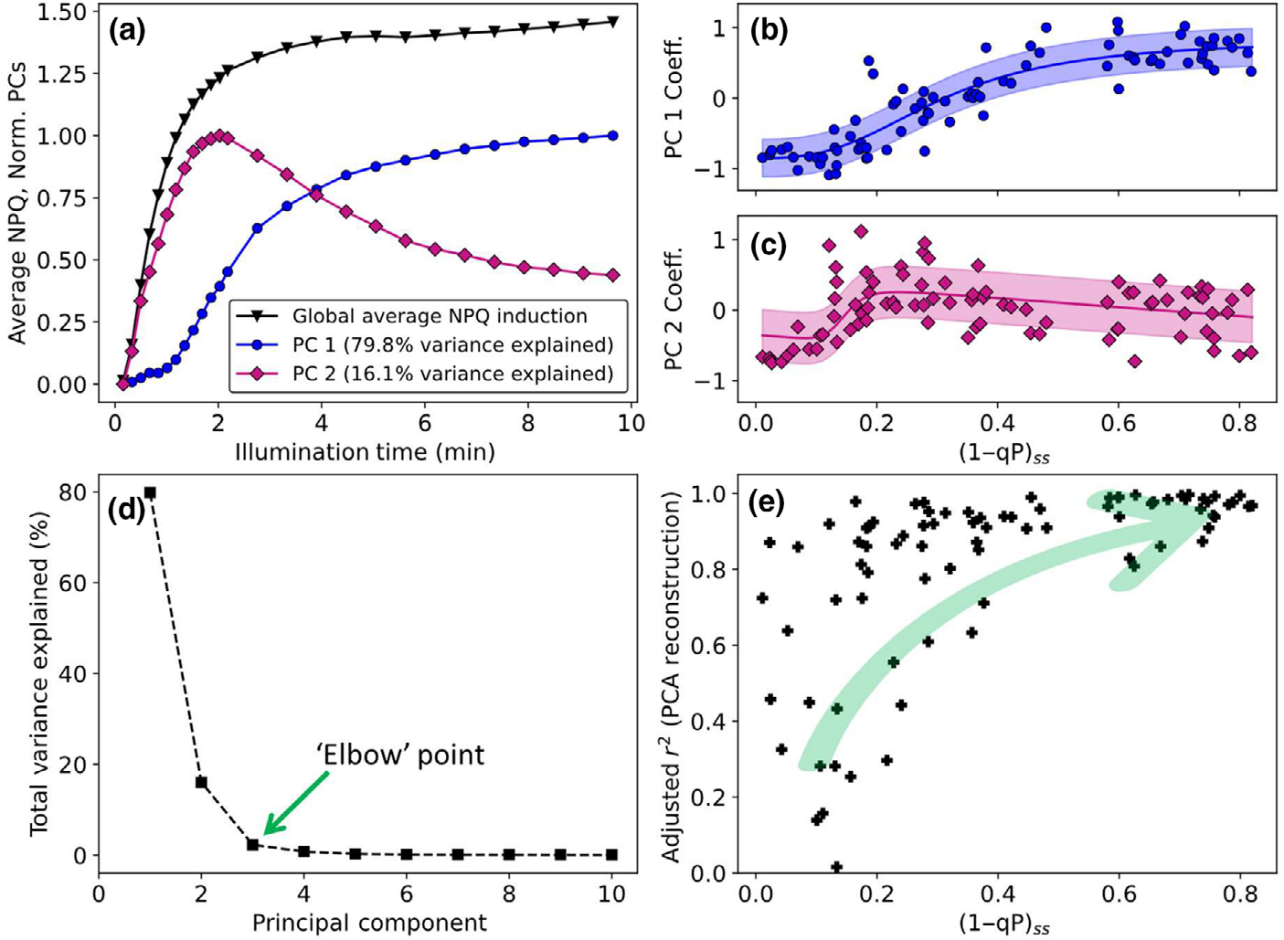
Summary of the first stage of the novel analysis pipeline as applied to wild‐type *Arabidopsis thaliana*. Principal component analysis (PCA) of wild‐type *A. thaliana* nonphotochemical quenching (NPQ) induction curves measured (using the high‐resolution pulse sequence) over a range of different actinic light intensities showing (a) the most important normalised principal components and their coefficients; (b) following a sigmoid with a turning point near (1−qP)_ss_ ≈ 0.3; (c) following a sloped sigmoid with a turning point at (1−qP)_ss_ ≈ 0.12 and a peak intensity at (1−qP)_ss_ ≈ 0.2, the shaded area shows the biological variance associated with each component; (d) associated scree plot (‘elbow’ point is indicated by a green arrow); and (e) Adjusted *r* ^2^ values obtained for the PCA reconstructions of the wild‐type *A. thaliana* NPQ induction data set.

To further explore the changes in the NPQ induction as the actinic light intensity increases, a data set consisting of 85 NPQ induction curves measured at different red actinic light (Fig. S1b) intensities was explored (hereafter referred to as the data set). This data set contained measurements performed on individual randomly selected leaves, obtained from a series of randomly selected wt *A. thaliana* plants to ensure that this data set also captured the biological variation known to be present between different leaves and plants. This data set was mean‐centred and analysed using PCA (Maćkiewicz & Ratajczak, 1993; Fritzsch *et al*., 2018) to explore the sources of variation between the individual NPQ induction curves. The results of this analysis are summarised in Fig. 2.

The PCA shows that the majority of the total variance within the data set can be well‐represented by just two principal components (PCs) which explain 79.8% (PC1, blue curve in Fig. 2a) and 16.1% (PC2, dark magenta curve in Fig. 2a) of the total variance, respectively (associated scree plot, Fig. 2d). The associated scree plot illustrates that the ‘elbow’ point is located at *n* = 3, further supporting that the variation in the data set is adequately described by the first two PCs. PC1 is associated with an induction curve which increases monotonically with illumination time, approaching a steady state after *c*. 6 min of illumination (blue curve in Fig. 2a). This behaviour is broadly similar to the NPQ induction curves measured at high (1−qP)_ss_ values ((1−qP)_ss_ > 0.5, Fig. 1). Contrasting this, PC2 (diamonds in Fig. 2a) shows a large increase in intensity, peaking at 2 min of illumination, before undergoing a gradual decrease to a lower steady‐state value. Interestingly; this is similar to the line shape of the induction curve seen at (1−qP)_ss_ = 0.125 (blue diamonds in Fig. 1).

**Fig. 3:**
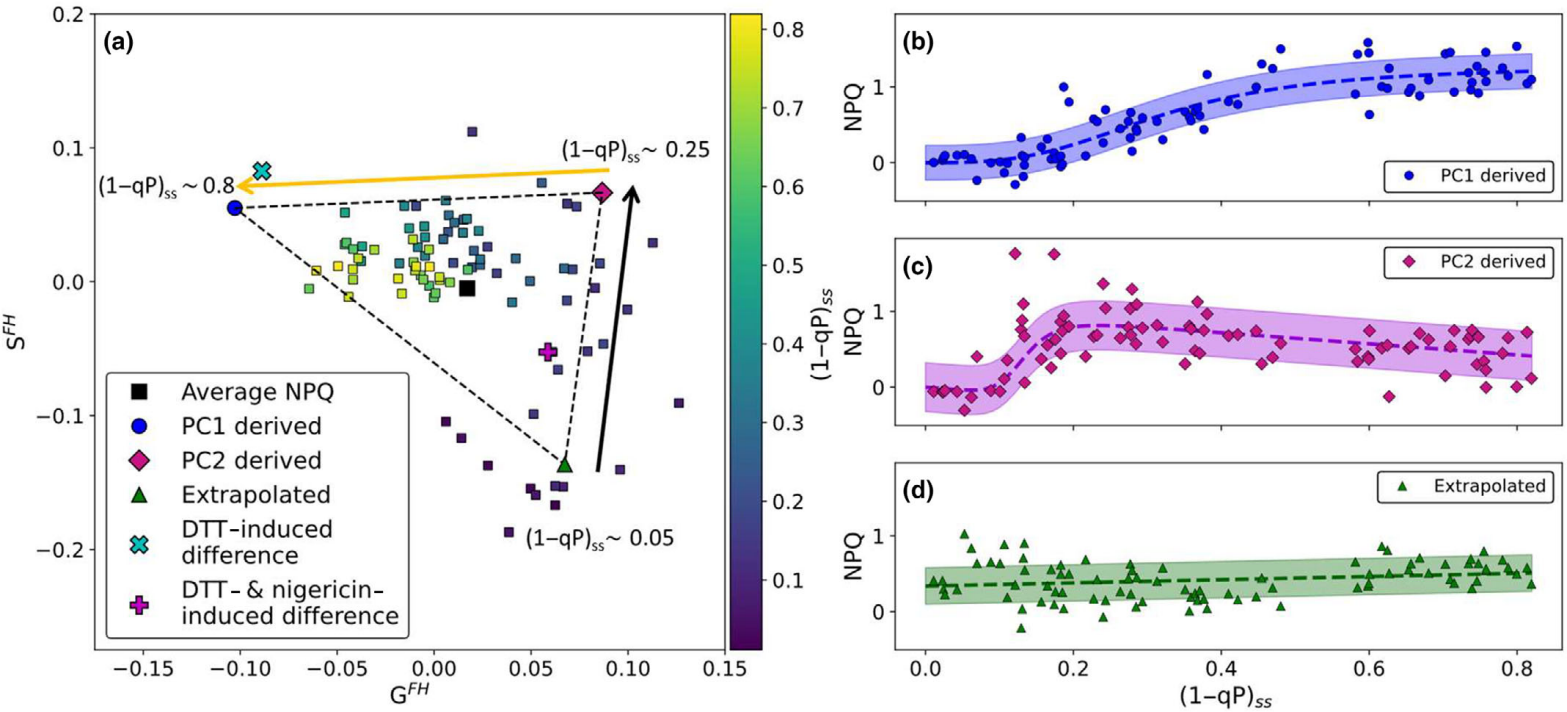
Summary of the second stage of the novel analysis pipeline as applied to wild‐type *Arabidopsis thaliana*. (a) Full harmonic phasor analysis (FH‐PhA) of wild‐type *A. thaliana* nonphotochemical quenching (NPQ) induction curves measured (using the high‐resolution pulse sequence) over a range of different actinic light intensities (from (1−qP)_ss_ ~ 0.05 (blue squares) to (1−qP)_ss_ ~ 0.8 (yellow squares)) as well as the phasor derived coefficients (the trajectory of the data through the full harmonic phasor space is shown by the black and yellow arrows) (b) following a sigmoid with a turning point at (1−qP)_ss_ ≈ 0.3, (c) following a sloped sigmoid with a turning point at (1−qP)_ss_ ≈ 0.12 and a peak intensity at (1−qP)_ss_ ≈ 0.2 and (d) linear with a gradient of 0.21 and an intercept of 0.34, the shaded area shows the biological variance associated with each component.

Additionally, PCA provides a set of coefficients associated with PC1 and PC2 (summarised in Fig. 2b,c, respectively), illustrating how these PCs contribute to the different induction curves within the data set with respect to the global mean (black, Fig. 2a). For PC1, these coefficients vary in a sigmoidal manner with increasing (1−qP)_ss_, with a turning point located at (1−qP)_ss_ = 0.3 (blue circles, Fig. 2b). Similarly, the PC2 coefficients also vary following a sigmoid with increasing (1−qP)_ss_; however, in this case, the turning point is located at (1−qP)_ss_ = 0.125 (magenta diamonds, Fig. 2c). PCA performed on the data set obtained using the low‐resolution pulse sequence yields very similar results, with the only difference being that the turning points are located at higher values of (1−qP)_ss_ (Fig. S6), suggesting the high‐resolution pulse sequence is actinic in nature. Together with the global average NPQ induction curve of the data set (black, Fig. 2a), these coefficients and their associated PCs can be used to reconstruct the original curves (several representative PCA reconstructions are shown in Fig. S7). Exploring the adjusted *r* ^2^ values of these PCA reconstructions, (Fig. 2e) shows that these reconstructions represent the data adequately for induction curves measured at (1−qP)_ss_ > 0.4; however, as (1−qP)_ss_ decreases below this value, the adjusted *r* ^2^ values rapidly decrease. This demonstrates that at lower actinic light intensities, the identified PCs no longer fully explain the NPQ induction curves within the data set. Taken together with the fact that PCA is specifically designed to extract sources of variation within the data, this decrease in the adjusted *r* ^2^ values (Fig. 2e) at low (1−qP)_ss_ values suggests the presence of a weakly varying component (with respect to the biological variation), which is dominant at low light intensities (Maćkiewicz & Ratajczak, 1993; Fritzsch *et al*., 2018). It is possible that such a component would be difficult to extract via PCA (due to the inherent ordering ambiguity of the PCs below the ‘elbow’ point, Fig. 2d) and so to investigate this possibility the data set was explored using a PCA‐guided FH‐PhA (Bader *et al*., 2014; Pârvu & Gilbert, 2016; Franssen *et al*., 2020; Torrado *et al*., 2022). The results of this analysis are summarised in Fig. 3.

FH‐PhA transforms each of the NPQ inductions curves into the associated 2D phasor space where the G^FH^‐ and S^FH^‐coordinate of the point reflect the line shape of the induction curve. Therefore, as the line shape of the NPQ induction curve changes, the associated position in this phasor space also changes. As previously noted as the (1−qP)_ss_ value increases, the NPQ induction curve line shape undergoes a series of nontrivial changes, which are reflected in the phasor space by a change in position as shown in Fig. 3(a). Specifically, NPQ induction curves leading to steady‐state (1−qP)_ss_ values ≤ 0.05 are observed to cluster around G^FH^ and S^FH^ coordinates of (0.05, −0.16), those corresponding to (1−qP)_ss_ ≈ 0.25 are located *c*. (0.08, 0.07) and finally curves leading to (1−qP)_ss_ values ≥ 0.75 are found near (−0.06, 0.04). As the value of (1−qP)_ss_ increases, the overall trajectory of the data in phasor space moves between these three positions following the black and yellow arrows in Fig. 3(a), respectively. Overall, these changes result in a data cloud, which is broadly triangular in shape (Fig. 3a). When the previously identified PCs are transformed into this phasor space, PC1 is located at (−0.1, 0.06) (blue circle, Fig. 3a) and PC2 is located at (0.09, 0.07) (magenta diamond, Fig. 3a). Importantly, these two points occur at two of the vertices of the triangular data cloud. Specifically, the point associated with PC2 is located near the (1−qP)_ss_ ≈ 0.25 vertex and the point arising from PC1 is located near the vertex associated with high values of (1−qP)_ss_. This is broadly consistent with the fact that these PCs only adequately describe the data at higher values of (1−qP)_ss_ (Fig. 2e).

The two vertices identified by the transformation of the PCs, combined with the data‐cloud centroid located at the position of the average NPQ induction curve (black square, Fig. 3a) allows the final vertex of the triangle to be calculated using Euclidian geometry (explanation in Fig. S8). This third, extrapolated vertex is found to be located near the point associated with low values of (1−qP)_ss_ (green triangle, Fig. 3a). Once these vertices have been identified, and assuming that the components represented by these vertices can fully represent the data, linear algebra is used to extract the relative abundances of these components underlying each of the induction curves in the data set (Franssen *et al*., 2020; Torrado *et al*., 2022). These contributions are shown in Fig. 3(b–d), respectively. The relative abundances of the vertices associated with the PCs (Fig. 3a–c) vary with (1−qP)_ss_ following sigmoidal curves, showing that the results of the FH‐PhA is fully consistent with PCA for these two components. Finally, the relative abundance associated with the extrapolated third component varies linearly with (1−qP)_ss_ (Fig. 3d). Interestingly, it is seen that this third component is dominant at low light intensities and varies relatively weakly with respect to the biological variation within the data set. Whilst the combination of PCA and FH‐PhA revealed in an unbiased way that the data set can be explained by three distinct components, it is important to note that these multivariate approaches yield approximate solutions and do not reveal the induction profiles of all three components.

**Fig. 4:**
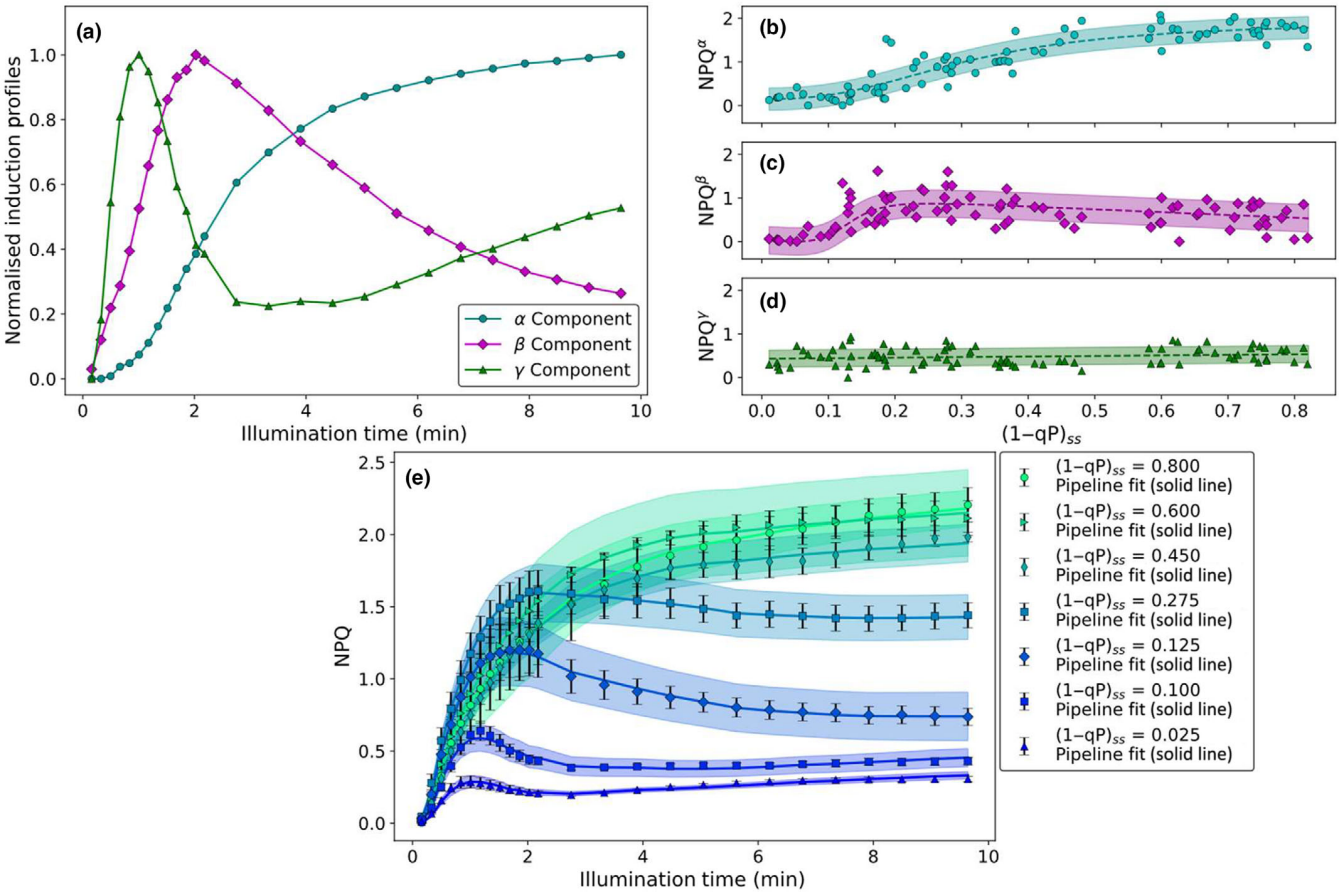
Summary of the third and final stage of the novel analysis pipeline as applied to wild‐type *Arabidopsis thaliana* alongside a comparison between the results of the pipeline and the original data set. The results of the final multivariate analysis‐guided non‐negative matrix factorisation of the wild‐type *A. thaliana* nonphotochemical quenching (NPQ) induction dataset (measured using the high‐resolution pulse sequence) showing (a) the normalised induction profiles of the different components underlying the NPQ induction curves and the NPQ intensities associated with (b) the α component (cyan circles, following a sigmoid with a turning point at (1−qP)_ss_ = (0.38 ± 0.02) and an amplitude of (2.1 ± 0.1)), (c) the β component (magenta diamonds, following a sloped sigmoid with a turning point located at (1−qP)_ss_ = (0.126 ± 0.003) and a final amplitude of (0.69 ± 0.04)) and (d) the γ component (green triangles, varying linearly with a gradient of (0.13 ± 0.09) and an intercept of (0.43 ± 0.04)); (e) representative overall fits obtained from the analytical pipeline for NPQ induction curves (error bars showing the measurement SE) at a range of different (1−qP)_ss_ values. All plants were dark adapted overnight before measurement and illuminated using red actinic light. The shaded area shows the associated biological variance.

In order to refine this analysis and obtain induction profiles for each of the identified components, the results of the PCA‐guided FH‐PhA were used as to perform NMF (Berry *et al*., 2007; Naik, 2008) on the data set. Utilising the relative abundances of the three phases identified by the combination of PCA and FH‐PhA (Fig. 3b–d) as initial conditions of the contributions of these components, NMF was performed to obtain the optimised induction profiles and contributions to the overall NPQ associated with each of the identified phases. The results of this analysis are summarised in Fig. 4.

Multivariate analysis‐guided NMF reveals the normalised induction profile of three distinct components underpinning all of the NPQ induction curves obtained for wt *A. thaliana* (Fig. 4a). The fastest of these components, γ, has a complicated profile, containing both a local maximum after 1 min and minimum at 3.3 min of illumination, respectively. Beyond this local minimum, the amount of quenching associated with this phase slowly increases again over the rest of the illumination period. The slower rising component, β, has a profile similar to PC2 (diamonds in Fig. 2a), whilst the slowest component, α, is similar to PC1 (circles in Fig. 2a). The analysis thus far shows that just three induction profiles can explain all the NPQ induction curves. Aiming to assign the components to different molecular mechanisms, we continued with chemical treatments. Leaves were infiltrated with DTT and nigericin. DTT is an inhibitor of the xanthophyll cycle (Neubauer, 1993), to pinpoint the effect of zeaxanthin formation (Fig. S9a,b). Nigericin is a pH‐uncoupler (Horton *et al*., 2000; Brooks *et al*., 2013), allowing us to eliminate the acidification of the thylakoid lumen. The difference profile between the untreated and DTT‐treated NPQ profile (Fig. S9b) is similar to α, indicating that this component is related to zeaxanthin formation. Adding nigericin on top of DTT results in a difference profile that resembles the β and γ components (Fig. S9c,d). Suggesting that these components are related to lumen acidification. In addition to these normalised induction profiles, the contributions of these components to the overall NPQ as a function of (1−qP)_ss_ value, are shown in Fig. 4(b–d). These contributions are similar to those obtained from FH‐PhA (Fig. 3b–d). Briefly, the contributions of the components with profiles similar to the PCs increase in a sigmoidal manner. The abundance of the fastest component (γ, Fig. 4a) varies linearly with (1−qP)_ss_. These normalised induction profiles and their associated contributions can be used to reconstruct the experimental data (several representative reconstructions are shown in Fig. 4e). The adjusted *r* ^2^ values obtained for these reconstructions are, apart from one outlier, above 0.8 (Fig. S10), indicating that the three identified components adequately represent the experimental data. Finally, performing NMF analysis on the low‐resolution data set yielded very similar profiles and contributions for the α, β and γ components (Fig. S11), with the turning points associated with the contributions of the β and γ components being noted to occur at higher values of (1−qP)_ss_, suggesting a slightly lowered lumen pH during the high‐resolution pulse sequence. However, this slightly lowered lumen pH does not affect the shape of the profiles.

### The npq1 NPQ induction data set

**Fig. 5:**
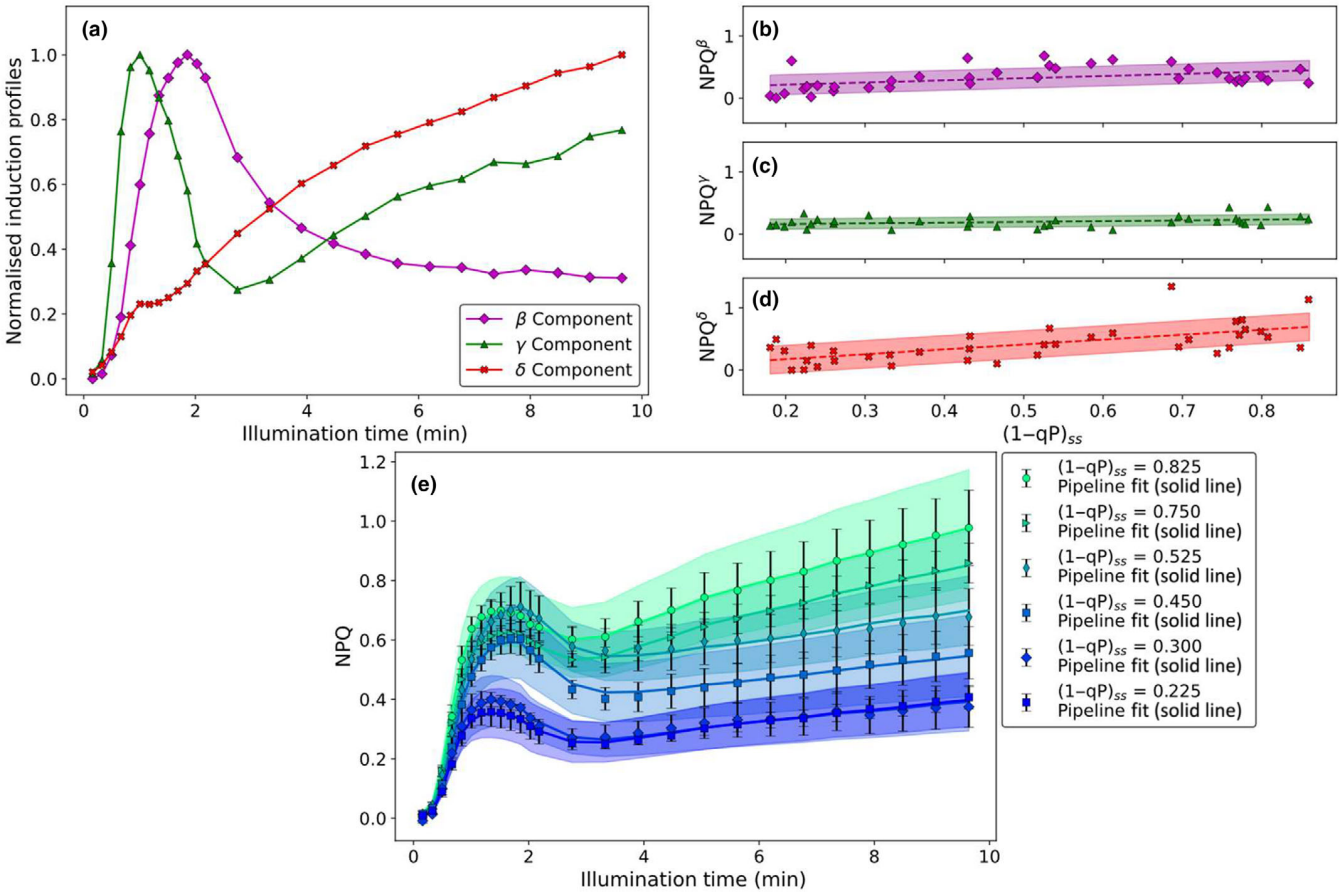
Summary of the third and final stage of the novel analysis pipeline as applied to *npq1 Arabidopsis thaliana* alongside a comparison between the results of the pipeline and the original data set. The results of multivariate analysis‐guided non‐negative matrix factorisation of the *npq1 A. thaliana* nonphotochemical quenching (NPQ) induction data set (measured using the high‐resolution pulse sequence) showing (a) the normalised induction profiles of the different components underlying the NPQ induction curves and the NPQ intensities associated with (b) the β component (magenta diamonds, linear with a gradient of (0.4 ± 0.1) and an intercept of (0.15 ± 0.07)), (c) the γ component (green triangles, linear with a gradient of (0.12 ± 0.06) and an intercept of (0.14 ± 0.03)) and (d) the δ component (red crosses, linear with a gradient of (0.8 ± 0. 2) and an intercept of (0.0 ± 0.1)); (e) representative overall fits obtained from the analytical pipeline for NPQ induction curves (error bars showing the measurement SE) at a range of different (1−qP)_ss_ values. All plants were dark adapted overnight before measurement and illuminated using red actinic light. The shaded area shows the associated biological variance.

Aiming to further elucidate the mechanisms underlying the NPQ components, the wt data were compared with the *npq1 A. thaliana* mutant. *Npq1* contains a nonfunctional version of VDE, meaning that it cannot accumulate zeaxanthin. Comparing the average NPQ induction curves of *npq1* (Fig. S12) and wt (Fig. 1) at a range of different (1−qP)_ss_ values reveals that this lack of zeaxanthin leads to significant changes in these curves. Specifically, the lack of zeaxanthin accumulation is reflected by the fact that the *npq1* NPQ induction curves have a distinct local maximum at all values of (1−qP)_ss_. Additionally, the overall amount of NPQ achieved by the *npq1* (Fig. S12) mutant is *c*. 60% lower than for wt (Fig. 1) after 10 min of illumination. Similar to the results seen in wt, (DTT & nigericin)‐treatment of *npq1* plants reveals a distinct induction profile associated with acidification of the lumenal space, which is thought to underly the induction curves in the *npq1* data set (Fig. S13). The multivariate NPQ deconvolution pipeline reveals that the induction curves in the *npq1* data set is underpinned by three distinct components (Fig. 5).

The fastest two of these components (β and γ, respectively, Fig. 5a) are similar to the fastest two components observed for the wt data set (Fig 4a, see Fig. S14 for a comparison). As for wt, the β‐component is similar to the (DTT & nigericin)‐induced difference curve (Fig. S13c) The line shape of the slowest component, δ, (crosses, Fig. 5a) is simply a gradual monotonic rise, and seemingly lacks any onset lag time, and is very different from the slowest component observed for wt *A. thaliana*. The contributions of these three components to the overall NPQ as a function of (1−P)_ss_ value are shown in Fig. 5(b–d). Briefly, the contributions of all of the identified components are found to vary linearly with (1−qP)_ss_. These normalised induction profiles and their associated contributions can be used to reconstruct the experimental data (several representative reconstructions are shown in Fig. S15a–e). The adjusted *r* ^2^ values obtained for these reconstructions are all above 0.8 for all explored (1−qP)_ss_ values (Fig. S15f), indicating that the three identified components adequately represent the experimental data.

## Discussion

### Exploring the *α* component

Whilst the multivariate analysis pipeline described here has revealed that all the induction curves in both of the data sets can be described by four components (α−δ), it cannot identify the molecular processes underpinning these components. Comparing these components with chemically induced NPQ difference curves allows these processes to be identified. First, the slowest component (α, circles in Fig. 4a) is similar to the DTT‐induced NPQ difference induction curve (Figs S9, S13) (Neubauer, 1993). Furthermore, previous studies utilising the light‐induced absorbance changes in the spectrum between 505 and 565 nm found induction profiles for the accumulation of zeaxanthin, which bear a strong resemblance to the monotonically increasing phase extracted from the wt NPQ induction data set (cyan, Fig. 4a) (Johnson *et al*., 2009). This identification is further supported by the fact that the phasor space location of the DTT‐induced NPQ difference induction curve (cyan triangle, Fig. 3a) is very close to PC1 (blue square, Fig. 3a) reflecting the fact that these two curves have very similar line shapes (Fig. S16a). Together, this demonstrates that this component reflects the accumulation of zeaxanthin during illumination (also referred to interchangeably as qZ or as part of qE).

**Fig. 6:**
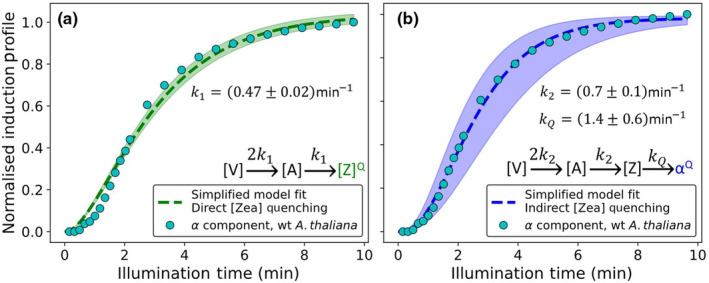
Overview of the fit to the α component (wild‐type *Arabidopsis thaliana*) of two simplified models describing the xanthophyll cycle. Comparison between a simplified model and the extracted normalised induction profile for (a) the *α* component (cyan circles), without a binding/interaction step (direct zeaxanthin quenching) and (b) the *α* component, with a binding/interaction step (indirect zeaxanthin quenching), extracted from the wild‐type *A. thaliana* data set. Inserts show the reactions explored in the simplified models and the shaded area shows the associated SE.

In order to explore whether the observed line shape can be explained by the molecular processes known to underpin the xanthophyll cycle, a simple model was constructed. Briefly, zeaxanthin is produced by the sequential de‐epoxidation of violaxanthin, by VDE. Since both of these de‐epoxidation steps involve the same enzyme and substrate moieties and violaxanthin is known to terminate with two identical epoxide moieties, it will be assumed that the rate of the formation of antheraxanthin is twice as high as the formation of zeaxanthin in this simplified model. Finally, the DTT‐induced differences in the post‐illumination NPQ recovery curves reveal that the zeaxanthin contribution to the NPQ relaxes faster than the measured epoxidation rates (Townsend *et al*., 2018) suggesting that this process involves an additional binding/interaction step (Fig. S17). To directly test this, both a direct zeaxanthin quenching (Fig. 6a) and an indirect zeaxanthin quenching version (Fig. 6b) of the model are considered. Interestingly, only the indirect zeaxanthin quenching model can fully explain the line‐shape of the *α* component, accurately recreating the sigmoidal line of this component including the initial onset lag.

### Exploring the *β* and *γ* components

The β and γ components (diamonds and triangles, Figs 4a, 5a) both exhibit a local NPQ maximum occurring after the first few minutes of illumination. Interestingly, this feature is also shared with the (DTT & nigericin)‐induced NPQ difference induction curve (Figs S9, S13), which was also found to exhibit a distinct local maximum within the first few minutes of illumination (Horton *et al*., 2000; Brooks *et al*., 2013). This suggests that this line shape arises from the establishment of the *trans*‐thylakoid pH difference during illumination. This conclusion is further supported by the phase space location of the (DTT and nigericin)‐induced NPQ difference induction curve (magenta triangles, Fig. 3a), which lies between the positions associated with the two remaining components (green triangle and magenta diamond, Figs 3a, S16a,b). This position suggests that the chemically induced difference curve is actually a linear combination of these two components (Fig. S15c,d). Furthermore, the line shapes of these components are also similar to the NPQ induction curves observed for the zeaxanthin‐lacking *npq1* mutant (Fig. S12) (Li *et al*., 2009). The chemical treatments (Figs S9, S13) suggest that these two components are thought to be consistent with the formation of a quenching species potentially associated with the protonation of PsbS due to the build‐up of a *trans*‐thylakoid pH difference (also referred to as qE). Interestingly, the differences in the line shapes of these two components (γ andβ, Fig. 4a) and their contribution (Fig. 4c,d) indicate that the characteristic induction profile of the protonation component changes as (1−qP)_ss_ increases, indicating the presence of biological processes that are modulated by the actinic light intensities.

### Exploring the *δ* component

Finally, the δ component is only found to significantly contribute to the *npq1* data set (Fig. 5a). This component follows a simple gradual monotonic rise, which starts as soon as the actinic light is turned on. The associated contribution to the NPQ (Fig. 5d) of this component varies linearly and passes through the origin. The presence of the β and γ components, and the fact that *npq1* is known to lack zeaxanthin suggests that this component cannot be explained as arising from either the xanthophyll cycle (like the α component in wt, circles Fig. 4a) or the protonation of PsbS (potentially like the β and γ components, diamonds and triangles in Figs 4a, 5a). The shape of the NPQ induction curve of the δ component is very similar to the induction curve observed for (DTT & nigericin)‐treated and DCMU‐treated plants (Fig. S18). These treatments are known to either eliminate (in the case of (DTT & nigericin)) or largely reduce (in the case of DCMU; which permanently closes PSII RCs) the production of the *trans*‐thylakoid membrane pH difference and, thus also prevents both the protonation of PsbS and the activation of VDE. Therefore, it is thought that this induced quenching can only be due to photoinhibition or qI. Finally, this assignment is further supported by the strong correlation seen between the amplitude of the δ component and the slowly reversible NPQ seen during recovery for the *npq1* leaves (Fig. S19).

### Conclusions

In conclusion, we have developed a novel multivariate analysis pipeline, which allows NPQ induction curves to be unravelled into their underlying components in an unbiased way. Applying this novel pipeline to the data sets of wt and *npq1 A. thaliana* NPQ induction curves revealed that all of these curves can be explained by four distinct components. Comparison with chemically treated leaves and data from literature allowed these different NPQ phases to be identified. The fastest two of these components, contributing to both the wt and *npq1* data sets (β and γ), were thought to arise from the protonation of PsbS driven by the accumulation of the *trans*‐thylakoid membrane pH difference (qE). In addition to these components, a third slower component (α) was found to be due to the accumulation of zeaxanthin and the subsequent activation of a quencher (the zeaxanthin contribution to qE/qZ). Finally, the *npq1* data revealed the slowest component (δ), which was thought to arise from qI‐like processes. Overall it seems that the α, β and γ components work in concert to provide an initial rapid high degree of protective quenching whilst the rest of the photosynthetic processes are still ramping up allowing the δ component (thought to be associated with qI) to be minimised. Overall, the novel pipeline reveals that the NPQ induction curves in wt and *npq1*, over a range of different actinic light intensities, can be explained by just four components, which arise from a few known underlying NPQ processes. Currently this approach is being expanded to allow the effects of mutations and other changes in the state (e.g. atmospheric composition or sequential illumination cycles) of the oxygenic photosynthetic organism to be directly probed in terms of changes in these underlying components.

## Competing interests

None declared.

## Author contributions

LAIR, EW and HA conceived the project. LAIR collected the data and performed the analysis. LAIR designed the experimental methodology and novel analysis pipeline, with substantial inputs from JH, EW and HA. LAIR and HA wrote the first draft of the manuscript with substantial inputs from JH and EW. All authors contributed to the manuscript.

## Supporting information


**Fig. S1** Normalised emission spectra of the lamps in the Hettich ESP PRC 1200 WL growth cabinet and the red actinic light source used in the pulse–amplitude–modulation measurements.
**Fig. S2** Summary of the non‐negative matrix factorisation algorithm, implemented in python3.9 (with comments).
**Fig. S3** Relationship between the applied actinic light intensity and (1–qP)_ss_, a proxy for the number of closed photosystem II reaction centres.
**Fig. S4** Overview of wild‐type *Arabidopsis thaliana* nonphotochemical quenching measured at different actinic light intensities with a low‐resolution saturating pulse sequence.
**Fig. S5** Comparison between the fits obtained from a simple linear‐combination model and nonphotochemical quenching induction curves at selected (1–qP)_ss_ values for wild‐type *Arabidopsis thaliana*, the adjusted *r* ^2^ values for the full data set are also included.
**Fig. S6** Summary of the first stage of the novel analysis pipeline as applied to the wild‐type *Arabidopsis thaliana* data set measured using the low‐resolution saturating pulse sequence.
**Fig. S7** Principal component analysis reconstructions of nonphotochemical quenching induction curves at selected (1–qP)_ss_ values for wild‐type *Arabidopsis thaliana*.
**Fig. S8** Explanation of how the third vertex of a triangle can be calculated for any triangle using the other two vertices and the centroid, with equations.
**Fig. S9** Summary of the effect of the d,l‐dithiothreitol and DTT & nigericin treatments on the nonphotochemical quenching of wild‐type *Arabidopsis thaliana* as well as the obtained differences in the nonphotochemical quenching induction curves.
**Fig. S10** Comparison between the fits obtained via the analysis pipeline and nonphotochemical quenching induction curves at selected (1–qP)_ss_ values for wild‐type *Arabidopsis thaliana*, the adjusted *r* ^2^ values for the full data set are also included.
**Fig. S11** Summary of the third and final stage of the novel analysis pipeline as applied to the wild‐type *Arabidopsis thaliana* data set measured using the low‐resolution saturating pulse sequence.
**Fig. S12** Overview of *npq1 Arabidopsis thaliana* nonphotochemical quenching measured at different actinic light intensities.
**Fig. S13** Summary of the effects of the d,l‐dithiothreitol and DTT & nigericin treatments on the nonphotochemical quenching of *npq1 Arabidopsis thaliana* as well as the obtained nonphotochemical quenching induction difference curve.
**Fig. S14** Comparison between the *β* and *γ* components identified for wild‐type and *npq1 Arabidopsis thaliana*.
**Fig. S15** Comparison between the fits obtained via the analysis pipeline and nonphotochemical quenching induction curves at selected (1–qP)_ss_ values for *npq1 Arabidopsis thaliana*, the adjusted *r* ^2^ values for the full data set are also included.
**Fig. S16** Comparison between the identified induction components and various chemically induced nonphotochemical quenching difference curves in full harmonic phasor space alongside the fits of the (d,ldithiothreitol & nigericin)‐induced nonphotochemical quenching difference curves for wild‐type and *npq1 Arabidopsis thaliana* using the *β*, *γ* and *δ* components.
**Fig. S17** Summary of the effect of d,l‐dithiothreitol treatment on the post‐illumination recovery of nonphotochemical quenching of wild‐type *Arabidopsis thaliana* as well as the obtained nonphotochemical quenching recovery difference curve.
**Fig. S18** Comparison between the nonphotochemical quenching of *npq1 Arabidopsis thaliana* following either 3‐(3,4‐dichlorophenyl)‐3,3‐dimethylurea or d,l‐dithiothreitol & nigericin treatment and the *npq1* δ component.
**Fig. S19** Nonphotochemical quenching (NPQ) data illustrating the rapidly and slowly reversible NPQ seen in *npq1 Arabidopsis thaliana* alongside a direct comparison between the size of the rapidly reversible NPQ and the sum of *β* and *γ* components as well as the size of the slowly reversible NPQ and the *δ* component.
**Notes S1** Equations and algorithms.
Please note: Wiley is not responsible for the content or functionality of any Supporting Information supplied by the authors. Any queries (other than missing material) should be directed to the *New Phytologist* Central Office.

## Data Availability

The original contributions presented in the study as well as the accompanying analysis pipeline software package (Yggdrasill_App.exe) are available to download from a Github repository: L‐Ramakers/Yggdrasill.
