# Supplementary material for: Unravelling the different components of nonphotochemical quenching using a novel analytical pipeline
Source: New Phytol. 2024 Nov 15;245(2):625–36. doi: 10.1111/nph.20271 (PMC11655427; doi:10.1111/nph.20271)
Supplement: Supplementary file 1 — Fig. S1 Normalised emission spectra of the lamps in the Hettich ESP PRC 1200 WL growth cabinet and the red actinic light source used in the pulse–amplitude–modulation measurements. Fig. S2 Summary of the non‐negative matrix factorisation algorithm, implemented in python3.9 (with comments). Fig. S3 Relationship between the applied actinic light intensity and (1–qP)ss, a proxy for the number of closed photosystem II reaction centres. Fig. S4 Overview of wild‐type Arabidopsis thaliana nonphotochemical quenching measured at different actinic light intensities with a low‐resolution saturating pulse sequence. Fig. S5 Comparison between the fits obtained from a simple linear‐combination model and nonphotochemical quenching induction curves at selected (1–qP)ss values for wild‐type Arabidopsis thaliana, the adjusted r 2 values for the full data set are also included. Fig. S6 Summary of the first stage of the novel analysis pipeline as applied to the wild‐type Arabidopsis thaliana data set measured using the low‐resolution saturating pulse sequence. Fig. S7 Principal component analysis reconstructions of nonphotochemical quenching induction curves at selected (1–qP)ss values for wild‐type Arabidopsis thaliana. Fig. S8 Explanation of how the third vertex of a triangle can be calculated for any triangle using the other two vertices and the centroid, with equations. Fig. S9 Summary of the effect of the d,l‐dithiothreitol and DTT & nigericin treatments on the nonphotochemical quenching of wild‐type Arabidopsis thaliana as well as the obtained differences in the nonphotochemical quenching induction curves. Fig. S10 Comparison between the fits obtained via the analysis pipeline and nonphotochemical quenching induction curves at selected (1–qP)ss values for wild‐type Arabidopsis thaliana, the adjusted r 2 values for the full data set are also included. Fig. S11 Summary of the third and final stage of the novel analysis pipeline as applied to the wild‐type Arabidopsis thaliana data [file NPH-245-625-s001.pdf]

New Phytologist Supporting Information

Article title: Unravelling the Different Components of Non-Photochemical Quenching using a novel Analytical Pipeline

Authors: Lennart A. I. Ramakers, Jeremy Harbinson, Emilie Wientjes and Herbert van Amerongen

Article acceptance date: 21 October 2024

The following Supporting Information is available for this article:

Figure S1: Normalised emission spectra of the lamps in the Hettich ESP PRC 1200 WL growth cabinet and the red actinic light (RAL) source used in the pulse-amplitude-modulation (PAM) measurements.

Figure S2: Summary of the non-negative matrix factorisation (NMF) algorithm, implemented in python3.9 (with comments).

Figure S3: Relationship between the applied actinic light intensity and $(1\text{-qP})_{ss}$, a proxy for the number of closed photosystem II (PSII) reaction centres.

Figure S4: Overview of wild-type *A. thaliana* non-photochemical quenching measured at different actinic light intensities with a low-resolution saturating pulse sequence.

Figure S5: Comparison between the fits obtained from a simple linear-combination model and non-photochemical quenching (NPQ) induction curves at selected $(1\text{-qP})_{ss}$ values for wild-type (wt) *A. thaliana*, the adjusted r^2 values for the full dataset are also included.

Figure S6: Summary of the first stage of the novel analysis pipeline as applied to the wild-type *A. thaliana* dataset measured using the low-resolution saturating pulse sequence.

Figure S7: Principal component analysis (PCA) reconstructions of non-photochemical quenching (NPQ) induction curves at selected $(1\text{-qP})_{ss}$ values for wild-type (wt) *A. thaliana*.

Figure S8: Explanation of how the third vertex of a triangle can be calculated for any triangle using the other two vertices and the centroid, with equations.

Figure S9: Summary of the effect of the D,L-dithiothreitol (DTT) and DTT & nigericin treatments on the non-photochemical quenching of wild-type (wt) *A. thaliana* as well as the obtained differences in the NPQ induction curves.

Figure S10: Comparison between the fits obtained *via* the analysis pipeline and non-photochemical quenching (NPQ) induction curves at selected $(1\text{-qP})_{ss}$ values for wild-type (wt) *A. thaliana*, the adjusted r^2 values for the full dataset are also included.

Figure S11: Summary of the third and final stage of the novel analysis pipeline as applied to the wild-type *A. thaliana* dataset measured using the low-resolution saturating pulse sequence.

Figure S12: Overview of *npq1 A. thaliana* non-photochemical quenching (NPQ) measured at different actinic light intensities.

Figure S13: Summary of the effects of the D,L-dithiothreitol (DTT) and DTT & nigericin treatments on the non-photochemical quenching (NPQ) of *npq1 A. thaliana* as well as the obtained NPQ induction difference curve.

Figure S14: Comparison between the β and γ components identified for wild-type and *npq1 A. thaliana*.

Figure S15: Comparison between the fits obtained *via* the analysis pipeline and non-photochemical quenching (NPQ) induction curves at selected $(1\text{-}qP)_{ss}$ values for *npq1 A. thaliana*, the adjusted r^2 values for the full dataset are also included.

Figure S16: Comparison between the identified induction components and various chemically induced non-photochemical quenching (NPQ) difference curves in full harmonic phasor space alongside the fits of the (D,L-dithiothreitol (DTT) & nigericin)-induced NPQ difference curves for wild-type (wt) and *npq1 A. thaliana* using the β, γ and δ components.

Figure S17: Summary of the effect of D,L-dithiothreitol (DTT) treatment on the post-illumination recovery of non-photochemical quenching (NPQ) of wild-type *A. thaliana* as well as the obtained NPQ recovery difference curve.

Figure S18: Comparison between the non-photochemical quenching (NPQ) of *npq1 A. thaliana* following either 3-(3,4-dichlorophenyl)-3,3-dimethylurea (DCMU) or D,L-dithiothreitol (DTT) & nigericin treatment and the *npq1* δ component.

Figure S19: Non-photochemical quenching (NPQ) data illustrating the rapidly and slowly reversible NPQ seen in *npq1 A. thaliana* alongside a direct comparison between the size of the rapidly reversible NPQ and the sum of β & γ components as well as the size of the slowly reversible NPQ and the δ component.

Notes on the equations underpinning full harmonic phasor analysis and the algorithm (python3.9) used to perform non-negative matrix factorisation (NMF) are also included.

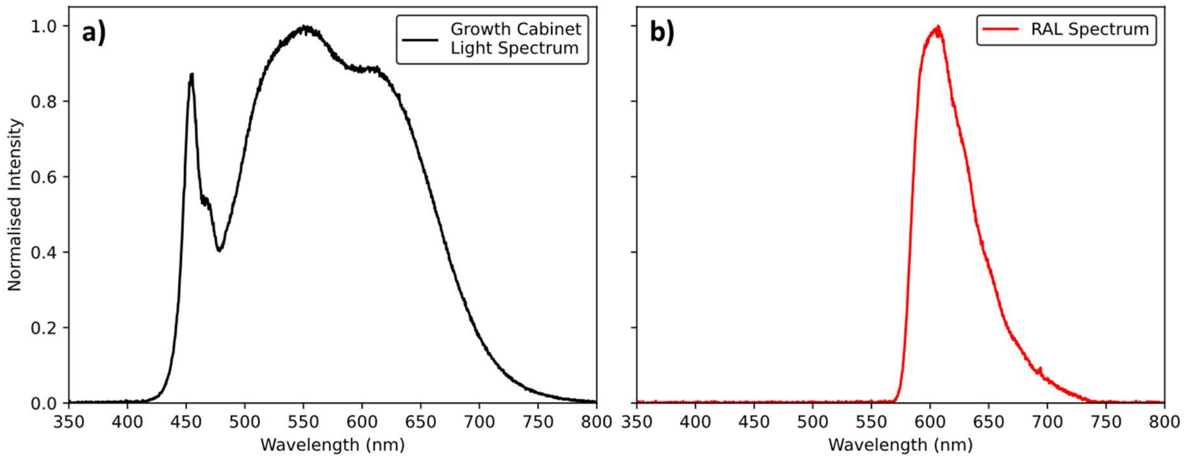

Figure S1: Normalised emission spectra of the lamps in the Hettich ESP PRC 1200 WL growth cabinet and the red actinic light (RAL) source used in the pulse-amplitude-modulation (PAM) measurements. Emission spectra of a) the actinic light utilised by the Hettich ESP PRC 1200 WL growth cabinet and b) the red actinic light used in the PAM measurements.

Notes S1 - *Equations and Algorithms:*

S1.1 – Full Harmonic Phasor Analysis (FH-PhA)

FH-PhA is an adapted form of standard phasor analysis (Bader et al., 2014; Pârvu and Gilbert, 2016; Franssen et al., 2020; Torrado, Malacrida and Ranjit, 2022). This method uses the standard harmonics of phasor analysis to maximise the differences between different line-shapes within any given dataset. This multivariate analysis can be performed using the following steps.

1) The allowed phasor harmonics are determined for the selected dataset using equation (1).

$$\omega_n = \frac{2n\pi}{T}, \qquad n = 1,2,3 \dots \quad (1)$$

In equation (1) ω_n is the frequency associated with each of the allowed phasor harmonics, T is the overall measurement time (in this case this is the total illumination time) and n is an integer (indicating the associated harmonic). The maximum allowed value of n is given by the Nyquist limit associated with the largest time step (in this case this is known to be 30s leading to a Nyquist limit of 1 Hz).

2) Once the allowed values of ω_n have been determined equations (2) and (3) are used to calculate the G_n and S_n for all of the identified phasor harmonics.

$$G_n = \frac{\int NPQ(t)\cos(\omega_n t)\,dt}{\int NPQ(t)dt} \quad (2)$$

$$S_n = \frac{\int NPQ(t)\sin(\omega_n t)\,dt}{\int NPQ(t)dt} \quad (3)$$

$$G^{FH} = \sum_i f_i G_i^{FH} \quad (4)$$

In equations (2) and (3) $NPQ(t)$ is the NPQ induction curve, G_n and S_n are the x- and y-coordinate of the induction curve in each harmonic of the phasor space and t is the time axis associated with the induction curve. From these equations it can be seen that each set of G_n and S_n calculated are simply the amplitudes of the real and imaginary components of the Fourier transform at each harmonic normalised to the induction curve.

3) PCA is then applied to the phasor coordinates calculated for the different phasor harmonics (ω_n). This PCA transforms the calculate harmonics into the full harmonic space (yielding G^{FH} (= PC 1) and S^{FH} (= PC 2)) and so maximises the difference between all the different possible line-shapes.
4) Each point in this full harmonic phasor space can be expressed as a linear combination of a small number of underlying components as shown in equations (4) and (5).

$$S^{FH} = \sum_i f_i S_i^{FH} \quad (5)$$

In equations (4) and (5) G^{FH} and S^{FH} are the coordinates of the measured NPQ induction curve. G_i^{FH} and S_i^{FH} are the phasor coordinates of the ith component (in this analytical pipeline these components are identified via PCA and extrapolation using geometric arguments as outlined in the results section for wt *A. thaliana*, Fig. 3.(a)). f_i is the fractional contribution of the ith component to the measured NPQ induction curve.

5) Combined with the fact that the sum of all of the fractional contributions yields unity ($\sum_i f_i = 1$), equations (4) and (5) can be solved to obtain the fractional contributions of each components for every NPQ induction curve in the dataset.
6) Finally, multiplying these factional contributions by the maximum NPQ obtained in each of the induction curves yields the relative contribution of each of the components over $(1\text{-qP})_{ss}$ (as seen in Fig. 3.(b),(c)&(d)).

S1.2 – Non-negative Matrix Factorisation (NMF) Python3.9 Algorithm

NMF is performed utilising an adapted form of the alternating least squares (ALS) algorithm (Berry et al., 2007; Naik, 2008). A commented copy of this algorithm is shown in Figure S2.

```python
n = len(NPQ_induction_time) # n is the length of the induction curve data e.g. the number of sat. pulses used.
k = NMF_input_data['FH-PhA Results - '+selected_con['current_selection']][1] # k is the number of components NMF will attempt to find (identified by PCA & FH-PhA).
maxinter = 10000 # This is the maximum number of iterations that the algorithm will run to attempt to find the components.
trace_AA = np.trace(np.matmul(data_to_analyse.T,data_to_analyse)) # This is the trace of the dataset, used to determine if the fit has converged.
limit = 1E-6 # This is the convergence limit.
H = np.random.random((k,n)) # The algorithm then sets up initially kinetics, these are random noise at this stage.
i = 0
while True:
    X = np.matmul(H,data_to_analyse.T)
    E = np.matmul(H,H.T)
    E_inv = np.linalg.inv(E)
    W = np.matmul(E_inv,X).T # The first four lines in this loop use linear algebra to calculate/update component contributions (W) using the kinetics (H) and the input data.
    W[W < 0] = 0 # Any negative numbers in W are set to zero.
    if i<11:
        W[:,n] = component_list[n][:,1] # For the first 11 iterations of the loop the calculated W are replaced by those identified by PCA & FH-PhA.
    O = np.matmul(W.T,W)
    O_inv = np.linalg.inv(O)
    P = np.matmul(W.T,data_to_analyse)
    H = np.matmul(O_inv,P) # The preceding 4 lines in this loop use linear algebra to calculate/update the kinetics (H) using the contributions (W) and the input data.
    H[H < 0] = 0 # Any negative numbers in H are set to zero.
    for n in range(k):
        H[n] = H[n]/max(H[n]) # The current kinetics (H) are normalised.
    i = i+1
    converge = trace_AA - 2*np.trace(np.matmul(H.T,P)) + np.trace(np.matmul(H.T,(np.matmul(O,H)))) # The convergence is checked using the current W and H.
    if i > 11:
        converge_m,converge_c = np.polyfit(np.asarray(np.arange(0.,6.1,1)),all_con[-7:],1)
        if i > maxinter or abs(converge_m) < limit:
            break # The preceding 4 lines check if there have been more than 11 iterations and if the convergence criterion has been met allowing the algorithm to finish.
```

Figure S2: Summary of the non-negative matrix factorisation (NMF) algorithm, implemented in python3.9 (with comments). Full commented python3.9 code showing the adapted alternating least squares (ALS) algorithm utilised in the analysis pipeline.

The input data (data_to_analyse) for algorithm is an array built up using the individual NPQ induction curves to be analysed, n is the number of saturating pulses used to measure the induction curves, k is the number of components to be considered (identified by PCA & FH-PhA) and component_list is a list of the component contributions (initial conditions as identified by PCA & FH-PhA).

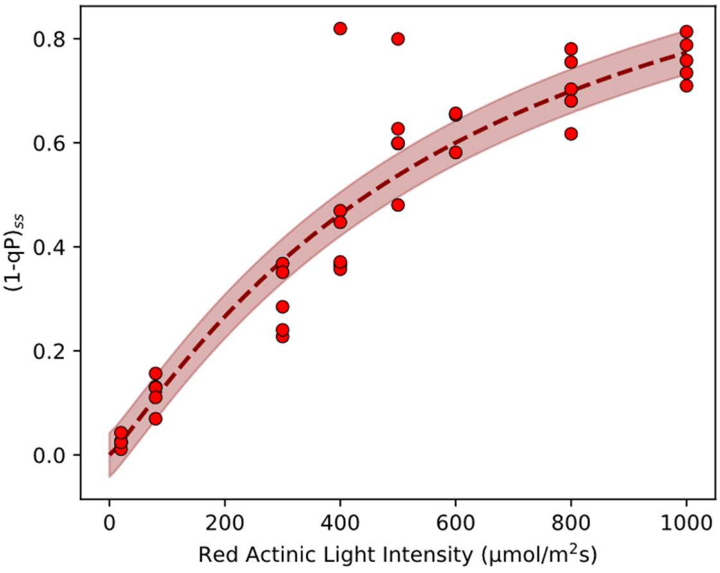

Figure S3: Relationship between the applied actinic light intensity and $(1\text{-}qP)_{SS}$, a proxy for the number of closed photosystem II (PSII) reaction centres. A proxy for the steady state fraction of closed PSII reaction centres $(1\text{-}qP)_{SS}$, after 10 minutes of illumination versus the intensity of the actinic light intensity for wild-type *A. thaliana*. The shaded area shows the associated standard error.

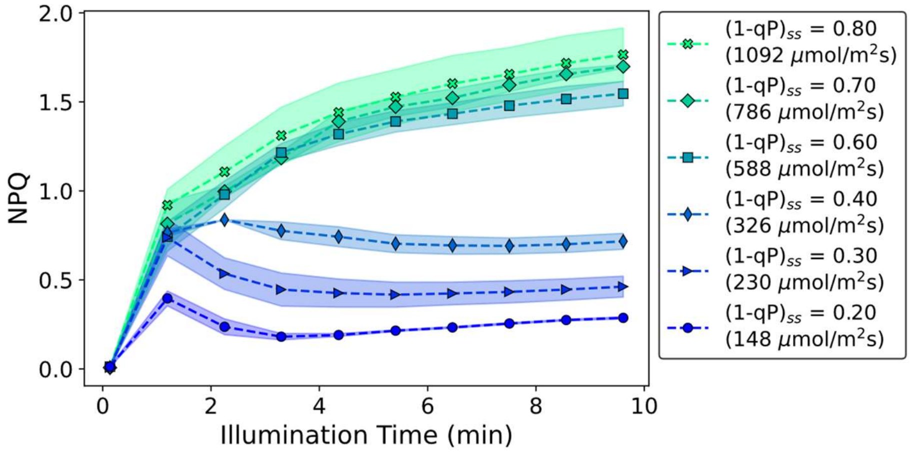

Figure S4: Overview of wild-type *A. thaliana* non-photochemical quenching measured at different actinic light intensities with a low-resolution saturating pulse sequence. Wild-type *A. thaliana* non-photochemical quenching (NPQ) induction curves obtained (with the low-resolution pulse sequence) at a range of different actinic light intensities, leading to differing values of $(1\text{-qP})_{ss}$. Each curve is the average of 5 individual measurements carried out on separate leaves, the shaded area shows the associated standard error.

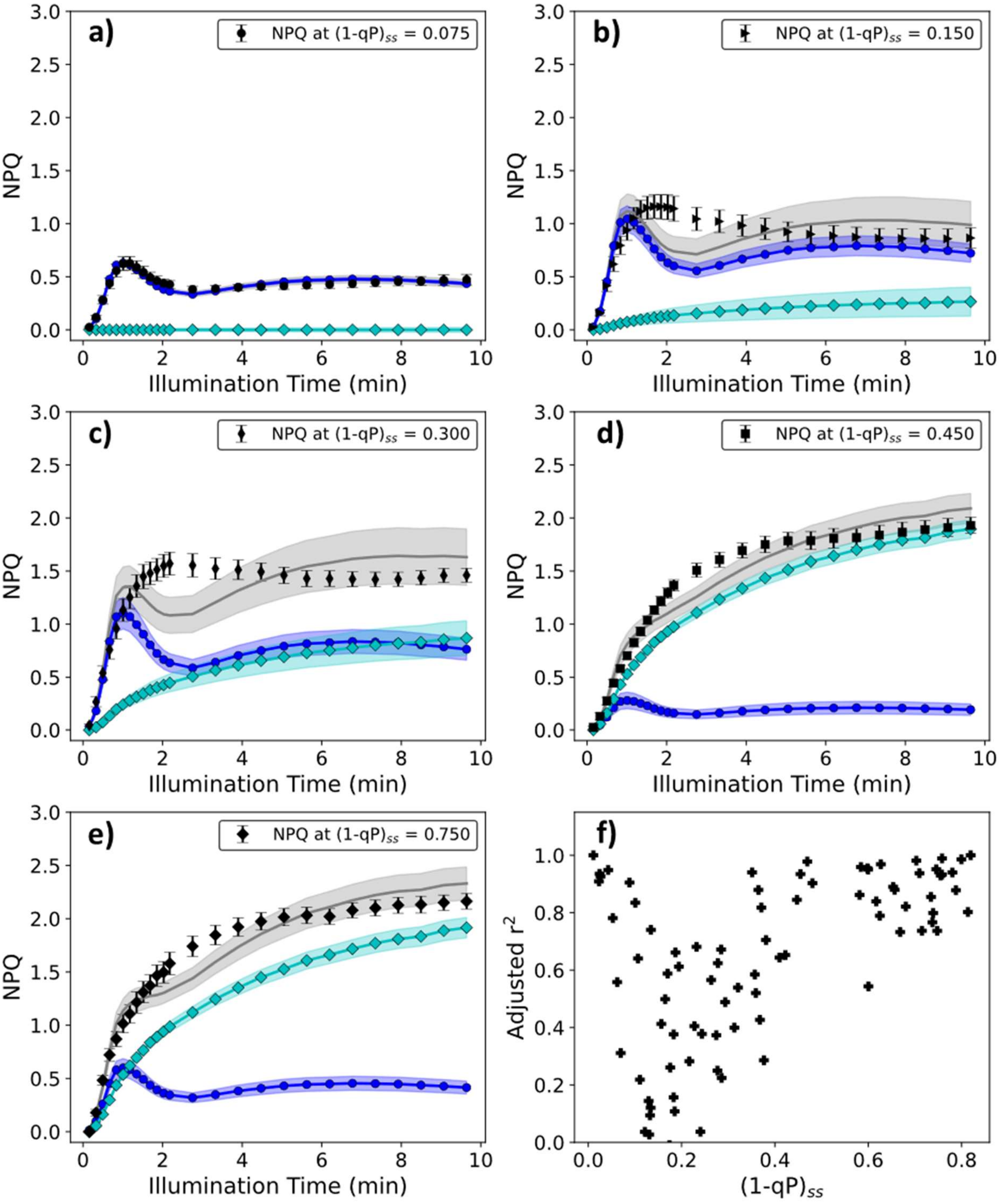

Figure S5: Comparison between the fits obtained from a simple linear-combination model and non-photochemical quenching (NPQ) induction curves at selected $(1\text{-qP})_{ss}$ values for wild-type (wt) *A. thaliana*, the adjusted r^2 values for the full dataset are also included. Representative fits (grey) of the NPQ induction curves (black) for wt *A. thaliana* at $(1\text{-qP})_{ss}$ values of a) 0.075, b) 0.15, c) 0.3, d) 0.45 and e) 0.75, obtained using the low light (blue, $(1\text{-qP})_{ss}$ = 0.02) and high light (blue-green, $(1\text{-qP})_{ss}$ = 0.81) NPQ induction curves, the shaded area shows the associated standard error; f) Adjusted r^2 values obtained for fitting the wt *A. thaliana* NPQ induction data set with the low light (blue circles, $(1\text{-qP})_{ss}$ = 0.02) and high light (cyan diamonds, $(1\text{-qP})_{ss}$ = 0.81) NPQ

induction curves. These representative fits demonstrate that this simplified linear-combination model cannot represent all of the changes seen in the data set.

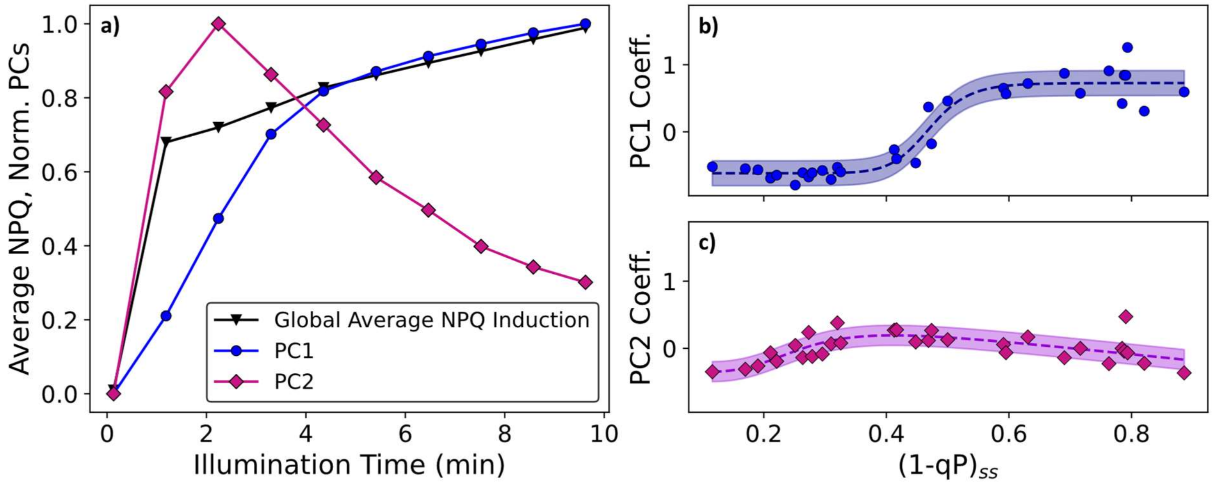

Figure S6: Summary of the first stage of the novel analysis pipeline as applied to the wild-type *A. thaliana* dataset measured using the low-resolution saturating pulse sequence. Principal component analysis (PCA) of wild-type *A. thaliana* non-photochemical quenching (NPQ) induction curves (obtained using the low-resolution sequence) measured over a range of different actinic light intensities showing a) the most important normalised principal components and their coefficients b) following a sigmoid with a turning point near $(1\text{-qP})_{ss} \approx 0.5$ & c) following a sloped sigmoid with a turning point at $(1\text{-qP})_{ss} \approx 0.25$ and a peak intensity at $(1\text{-qP})_{ss} \approx 0.2$, the shaded area shows the biological variance associated with each component.

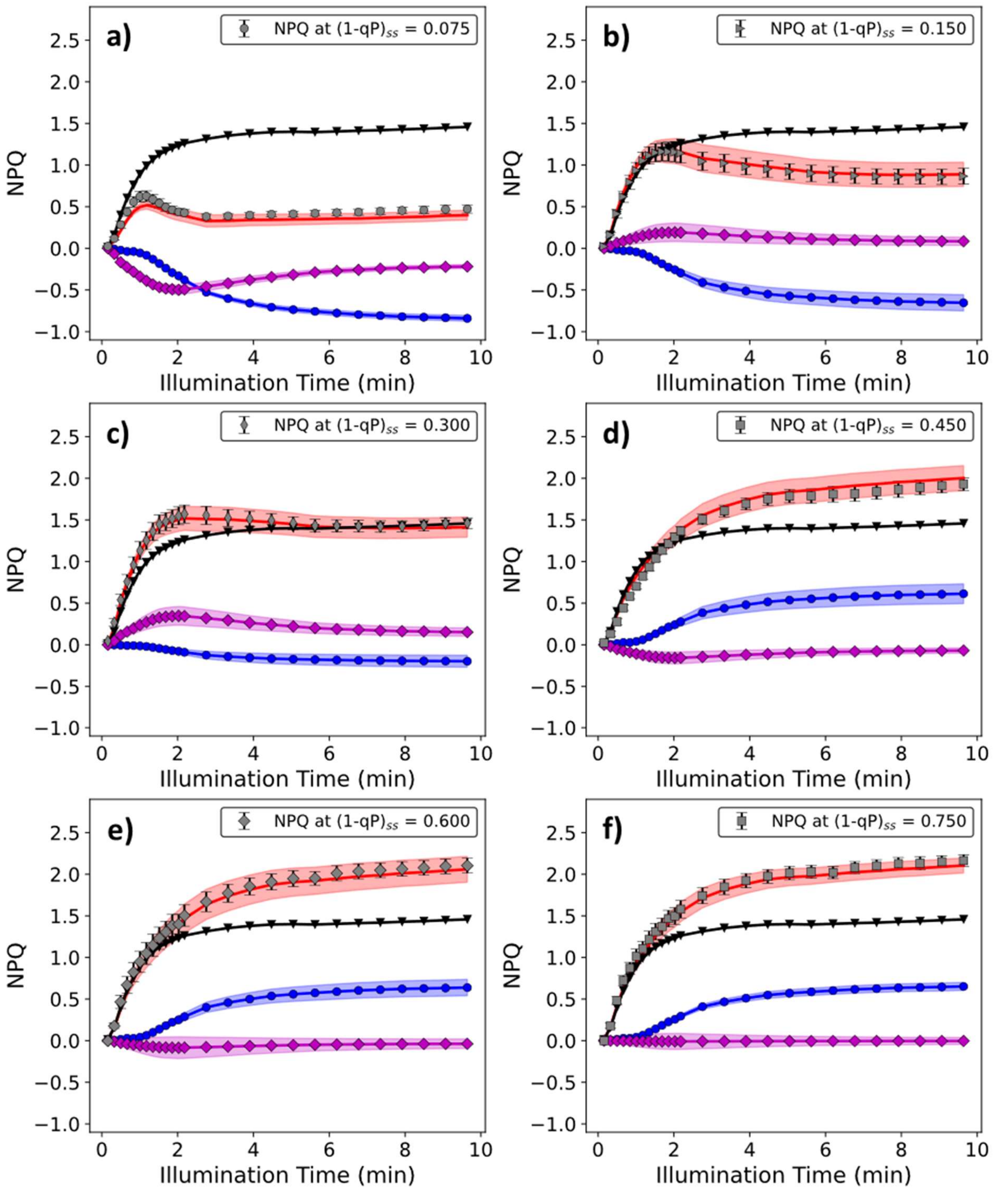

Figure S7: Principal component analysis (PCA) reconstructions of non-photochemical quenching (NPQ) induction curves at selected $(1\text{-}qP)_{ss}$ values for wild-type (wt) *A. thaliana*. Representative PCA reconstructions (red) of the NPQ induction curves (grey) for wt *A. thaliana* at $(1\text{-}qP)_{ss}$ values of a) 0.075, b) 0.15, c) 0.3, d) 0.45, e) 0.6 and f) 0.75, obtained using the average NPQ induction curve (black triangles), principal component 1 (PC1) (blue circles) and PC2 (purple diamonds). The shaded area shows the associated biological variance.

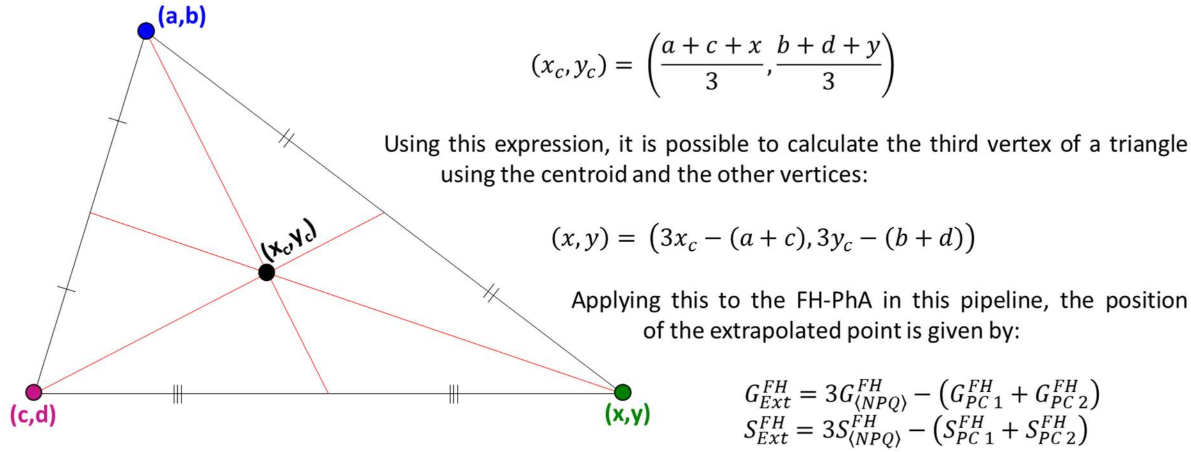

The centroid of any triangle with vertices located at **(a,b)**, **(c,d)** and **(x,y)** is defined as:

$$(x_c, y_c) = \left(\frac{a+c+x}{3}, \frac{b+d+y}{3}\right)$$

Using this expression, it is possible to calculate the third vertex of a triangle using the centroid and the other vertices:

$$(x, y) = \left(3x_c - (a+c), 3y_c - (b+d)\right)$$

Applying this to the FH-PhA in this pipeline, the position of the extrapolated point is given by:

$$G_{Ext}^{FH} = 3G_{\langle NPQ \rangle}^{FH} - \left(G_{PC\,1}^{FH} + G_{PC\,2}^{FH}\right)$$
$$S_{Ext}^{FH} = 3S_{\langle NPQ \rangle}^{FH} - \left(S_{PC\,1}^{FH} + S_{PC\,2}^{FH}\right)$$

Figure S8: Explanation of how the third vertex of a triangle can be calculated for any triangle using the other two vertices and the centroid, with equations. Diagram outlining how the third vertex of a triangle can be calculated using the centroid and the remaining vertices, using Euclidian geometry.

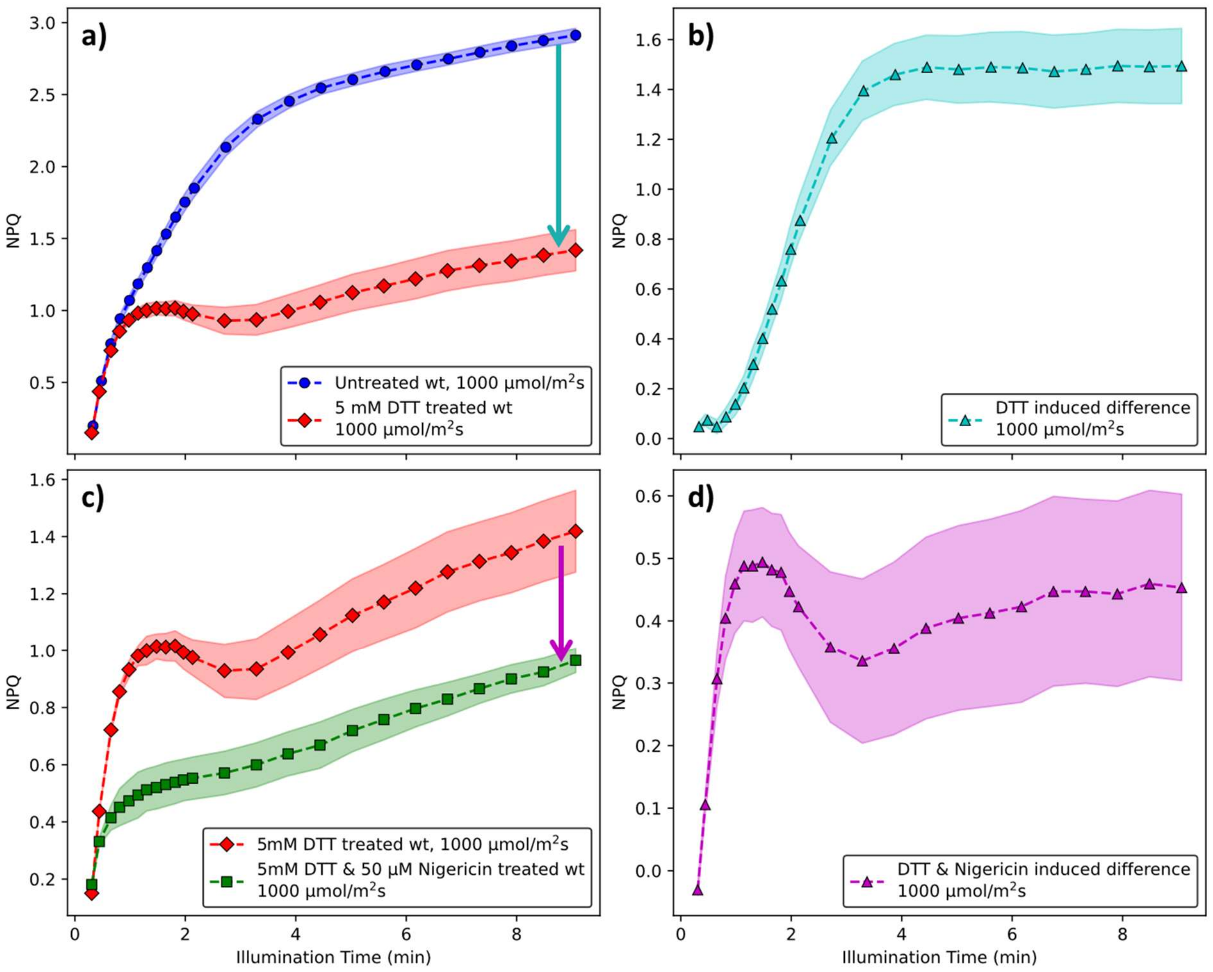

Figure S9: Summary of the effect of the D,L-dithiothreitol (DTT) and DTT & nigericin treatments on the non-photochemical quenching of wild-type (wt) *A. thaliana* as well as the obtained differences in the NPQ induction curves. a) NPQ induction curves for untreated (blue) and 5 mM DTT treated (red) wt *A. thaliana* leaves, recorded at an actinic light intensity of 1000 μmol/m^{2}s (the chemically induced difference is indicated with an arrow) and

b) the DTT-induced difference in the NPQ induction curve (cyan); c) NPQ induction curves for 5 mM DTT treated (red) and 5 mM DTT & 50 μM nigericin (green, residual NPQ thought to be due to a combination of imperfect chemical infiltration and the presence of photo-inhibitory processes enhanced by the chemical suppression of the other NPQ processes), recorded at an actinic light intensity of 1000 μmol/m^{2}s (the chemically induced difference is indicated with an arrow) and d) the (DTT & nigericin)-induced difference in the NPQ induction curve (magenta). All plants were dark adapted overnight prior to measurement and illuminated using red actinic light. The shaded area shows the associated standard error.

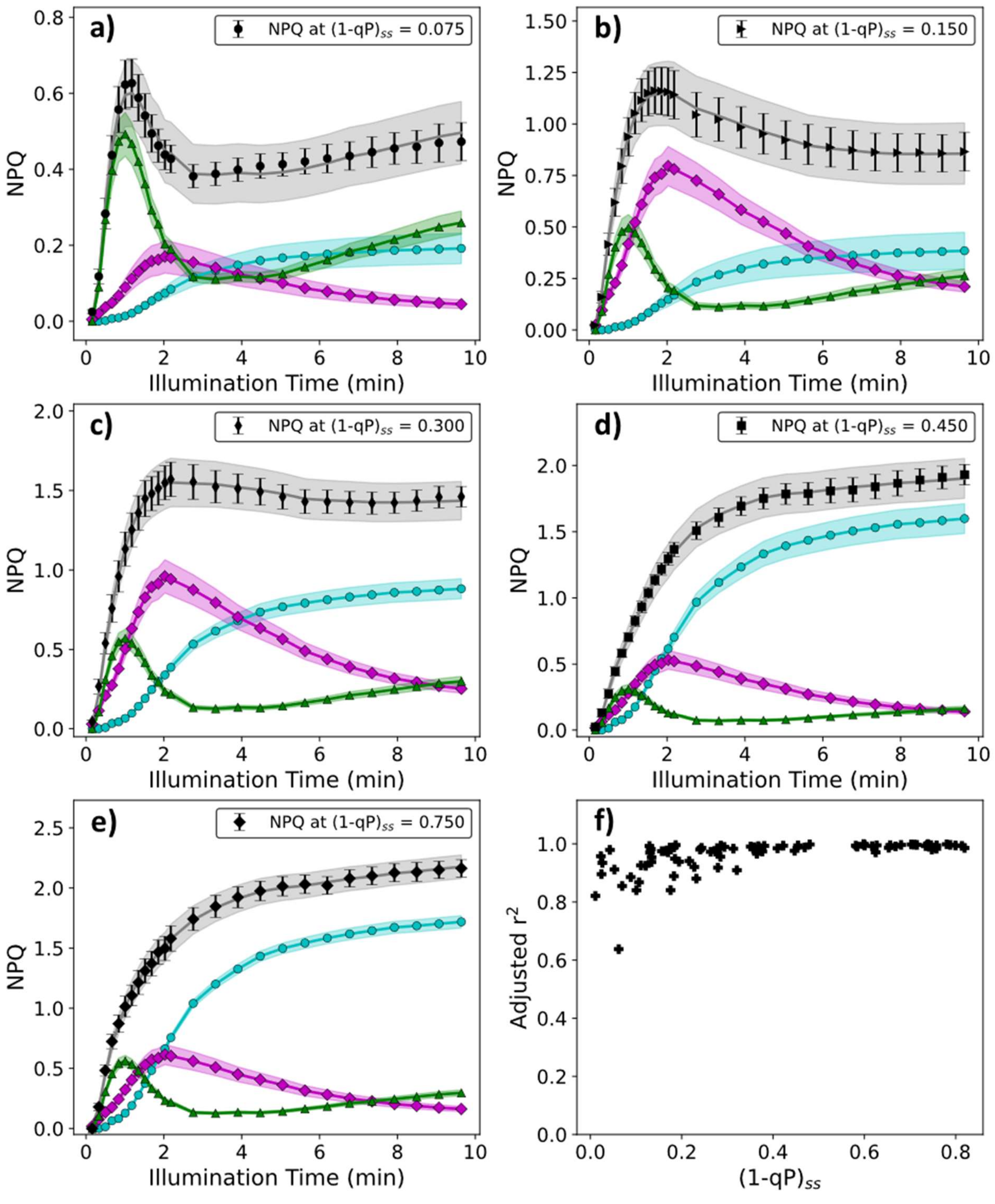

Figure S10: Comparison between the fits obtained *via* the analysis pipeline and non-photochemical quenching (NPQ) induction curves at selected $(1\text{-qP})_{ss}$ values for wild-type (wt) *A. thaliana*, the adjusted r^2 values for the full dataset are also included. Representative fits (grey) of the NPQ induction curves (black) for wt *A. thaliana* at $(1\text{-qP})_{ss}$ values of a) 0.075, b) 0.15, c) 0.3, d) 0.45 and e) 0.75, obtained using the α (cyan circles), β (magenta diamonds) and γ (green triangles) NPQ components; f) Adjusted r^2 values obtained for fitting the wt *A. thaliana* NPQ induction data set with the α (cyan circles), β (magenta diamonds) and γ (green triangles) NPQ components. The shaded area shows the associated biological variance.

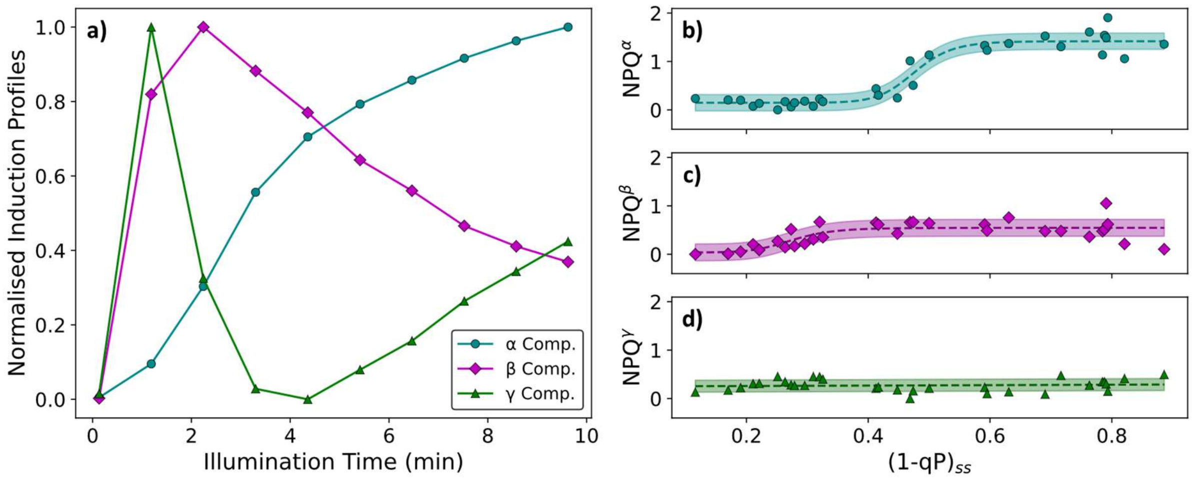

Figure S11: Summary of the third and final stage of the novel analysis pipeline as applied to the wild-type *A. thaliana* dataset measured using the low-resolution saturating pulse sequence. The results of the final multivariate analysis guided non-negative matrix factorisation of the wild-type *A. thaliana* non-photochemical quenching (NPQ) induction dataset (obtained using the low-resolution pulse sequence) showing a) the normalised induction profiles of the different components underlying the NPQ induction curves and the NPQ intensities associated with b) the α component (cyan circles, following a sigmoid with a turning point at $(1\text{-qP})_{ss}$ = (0.48±0.01) and an amplitude of (1.42±0.1)), c) the β component (magenta diamonds, following a sloped sigmoid with a turning point located at $(1\text{-qP})_{ss}$ = (0.28±0.03) and a final amplitude of (0.5±0.1)) and d) the γ component (green triangles, varying linearly with a gradient of (0.04±0.09) and an intercept of (0.25±0.05)). All plants were dark adapted overnight prior to measurement and illuminated using red actinic light. The shaded area shows the associated biological variance.

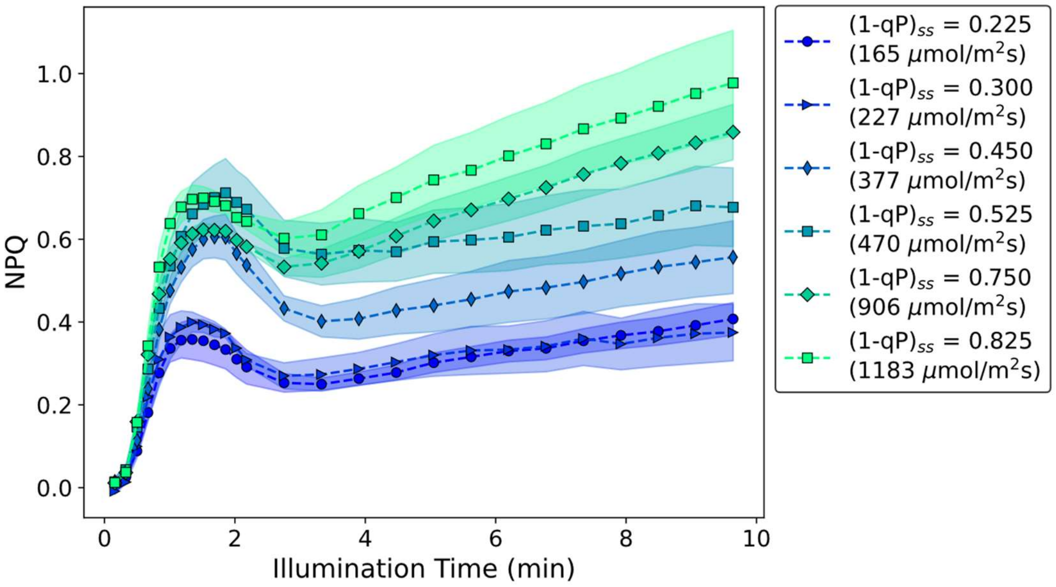

Figure S12: Overview of *npq1 A. thaliana* non-photochemical quenching (NPQ) measured at different actinic light intensities. *npq1 A. thaliana* NPQ induction curves obtained at a range of different actinic light intensities, leading to differing values of $(1\text{-qP})_{ss}$. Each curve is the average of 5 individual measurements carried out on separate leaves and the shaded area shows the associated standard error.

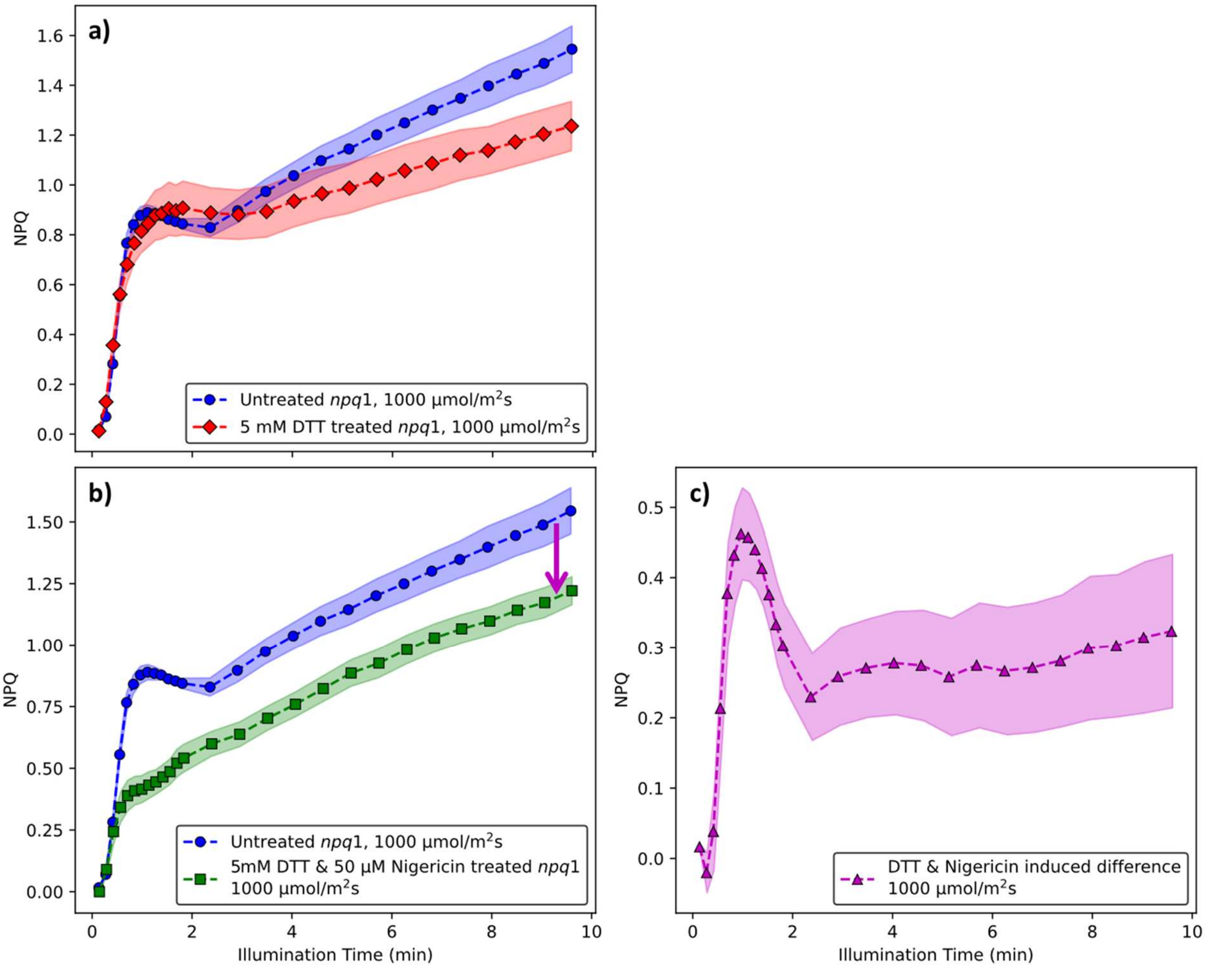

Figure S13: Summary of the effects of the D,L-dithiothreitol (DTT) and DTT & nigericin treatments on the non-photochemical quenching (NPQ) of *npq1 A. thaliana* as well as the obtained NPQ induction difference curve. a) NPQ induction curves for untreated (blue circles) and 5 mM DTT treated (red diamonds) *npq1 A. thaliana* leaves, recorded at an actinic light (AL) intensity of 1000 µmol/m^{2}s; b) NPQ induction curves for untreated (blue circles) and 5 mM DTT & 50 µM nigericin (green squares, residual NPQ due to imperfect chemical infiltration and the presence of photo-inhibitory processes enhanced by the chemical suppression of the other NPQ processes), recorded at an AL intensity of 1000 µmol/m^{2}s (the chemically induced difference is indicated with an arrow) and c) the 5 mM DTT & 50 µM nigericin induced difference in the NPQ induction curve (magenta triangles). All plants were dark adapted overnight prior to measurement and illuminated using red actinic light. The shaded area shows the associated standard error.

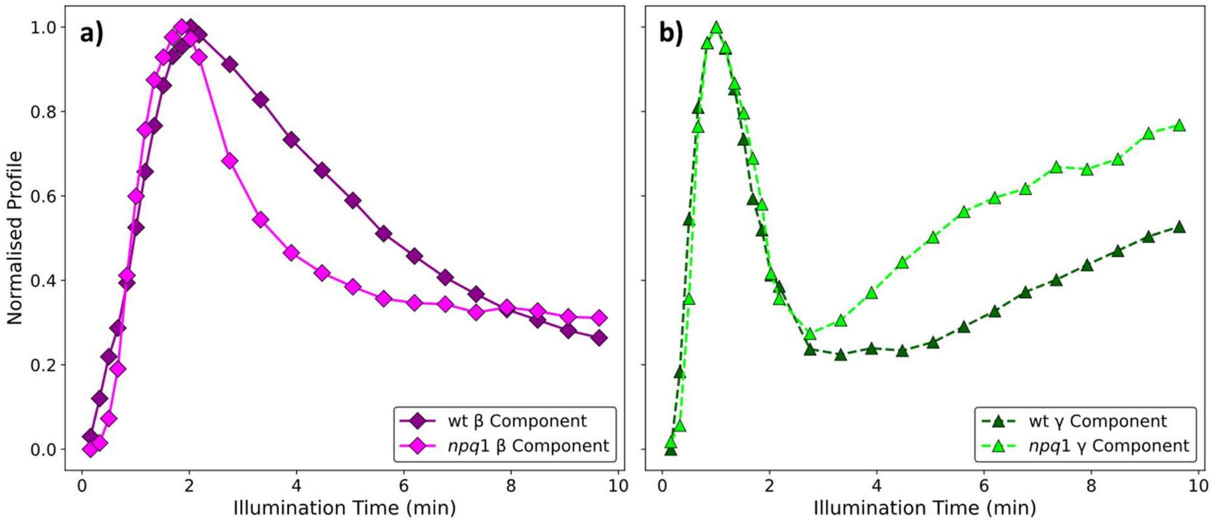

Figure S14: Comparison between the β and γ components identified for wild-type and *npq1 A. thaliana*. Comparison between the a) β and b) γ components identified from the wild-type and *npq1 A. thaliana* non-photochemical quenching (NPQ) induction data sets. The differences between the components for wt and *npq1* are thought to arise in part from compensatory effects due to the presence of the mutation.

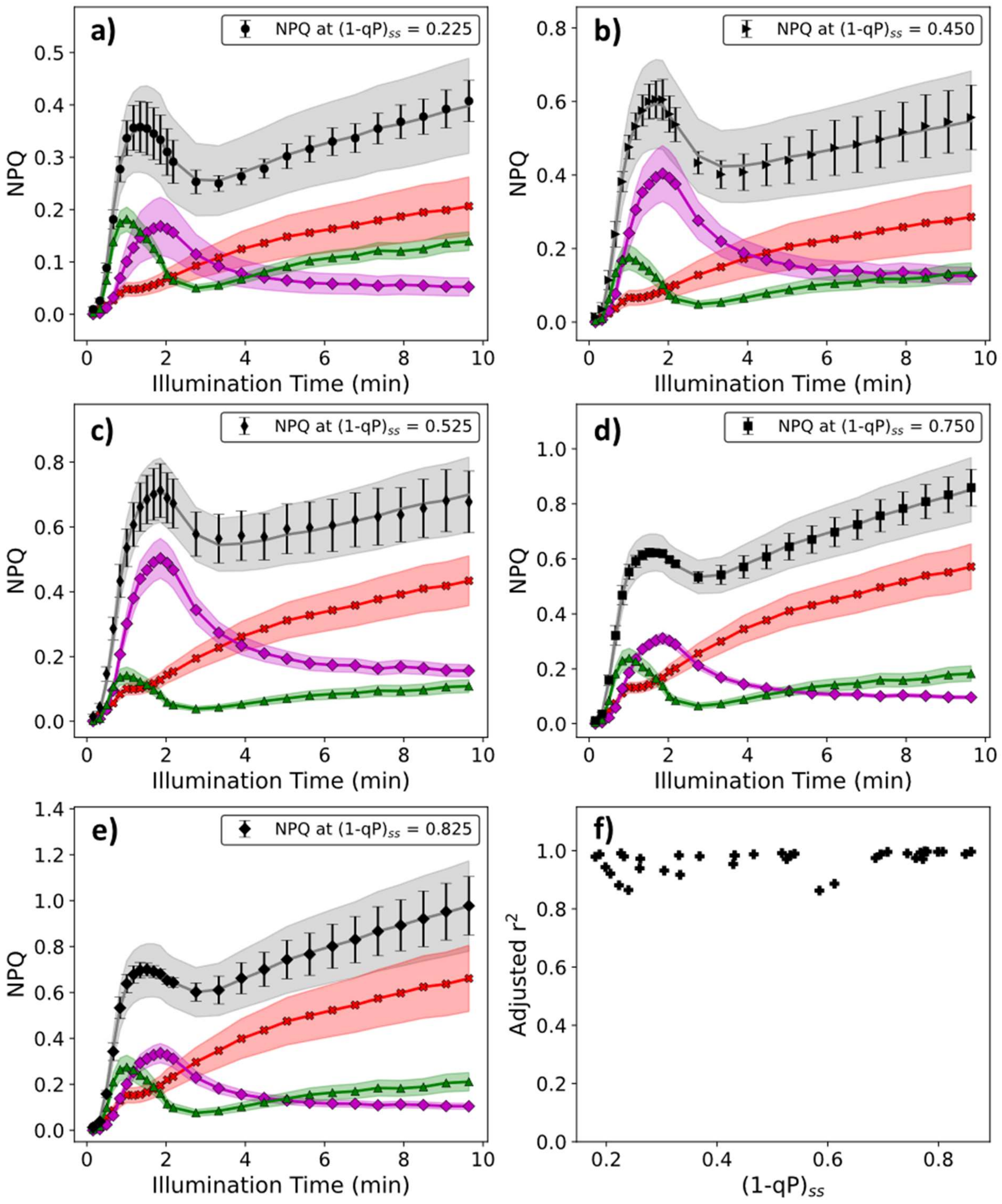

Figure S15: Comparison between the fits obtained *via* the analysis pipeline and non-photochemical quenching (NPQ) induction curves at selected $(1\text{-qP})_{ss}$ values for *npq1 A. thaliana*, the adjusted r^2 values for the full dataset are also included. Representative fits (grey) of the NPQ induction curves (black) for *npq1 A. thaliana* at $(1\text{-qP})_{ss}$ values of a) 0.225, b) 0.45, c) 0.525, d) 0.75 and e) 0.825, obtained using the β (magenta diamonds), γ (green triangles) and δ (red crosses) NPQ components; f) Adjusted r^2 values obtained for fitting the *npq1 A. thaliana* NPQ induction data set with the β (magenta diamonds), γ (green triangles) and δ (red crosses) NPQ components. The shaded area shows the associated biological variance.

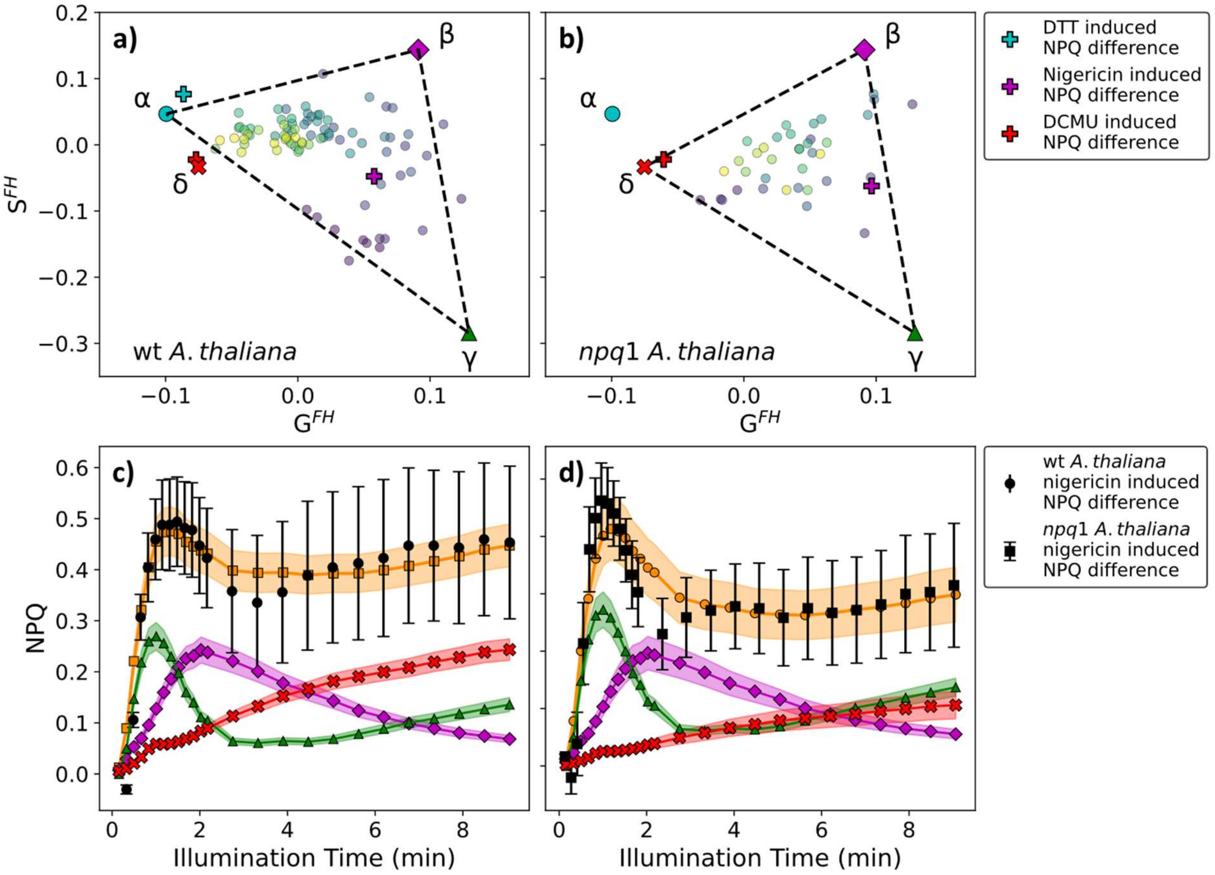

Figure S16: Comparison between the identified induction components and various chemically induced non-photochemical quenching (NPQ) difference curves in full harmonic phasor space alongside the fits of the (D,L-dithiothreitol (DTT) & nigericin)-induced NPQ difference curves for wild-type (wt) and *npq1 A. thaliana* using the β, γ and δ components. Full harmonic phasor analysis (FH-PhA) of the α (cyan circle), β (magenta diamond), γ (green triangle) and δ (red cross) components super-imposed over the a) wild-type and b) *npq1 A. thaliana* NPQ induction data sets; the positions of the DTT (cyan plus), (DTT & nigericin) (magenta plus) and 3-(3,4-dichlorophenyl)-3,3-dimethylurea (DCMU) (red plus) chemical treatments are also shown. Representative fits (orange circles) of the (DTT & nigericin)-induced NPQ difference curves obtained for c) wt and d) *npq1 A. thaliana* with the β (magenta diamonds), γ (green triangles) and δ (red crosses) components. The shaded area shows the associated biological variance.

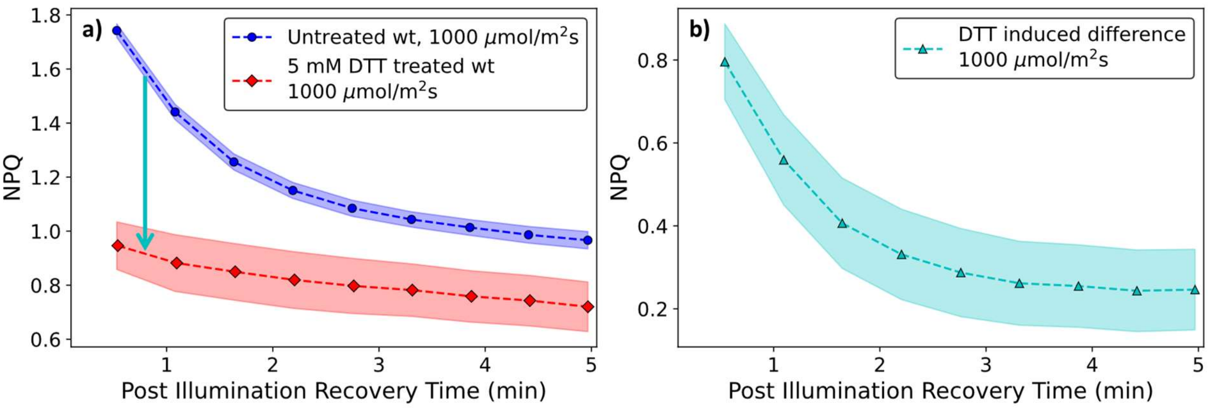

Figure S17: Summary of the effect of D,L-dithiothreitol (DTT) treatment on the post-illumination recovery of non-photochemical quenching (NPQ) of wild-type *A. thaliana* as well as the obtained NPQ recovery difference curve. a) NPQ post-illumination recovery curves for untreated (blue circles) and 5 mM DTT treated (red diamonds) wild-type *A. thaliana* leaves, recorded after illumination at an actinic light intensity of 1000 µmol/m^{2}s (the chemically induced difference is indicated with an arrow) and b) the 5 mM DTT induced difference in the NPQ post illumination recovery curve (cyan triangles). All plants were dark adapted overnight prior to measurement and illuminated using red actinic light. The shaded area shows the associated standard error.

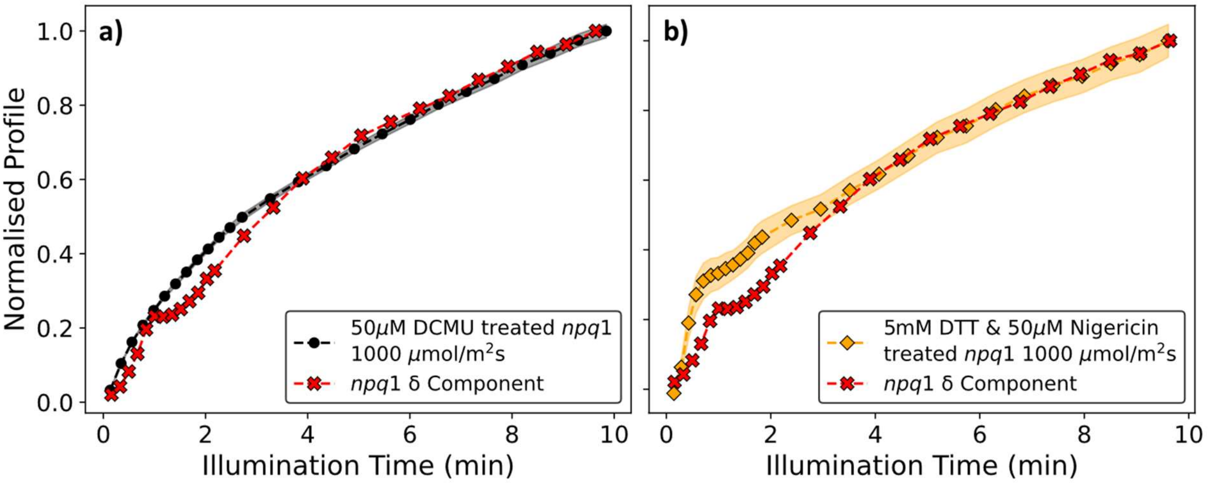

Figure S18: Comparison between the non-photochemical quenching (NPQ) of *npq1 A. thaliana* following either 3-(3,4-dichlorophenyl)-3,3-dimethylurea (DCMU) or D,L-dithiothreitol (DTT) & nigericin treatment and the *npq1* δ component. a) NPQ induction curves for 50 µM DCMU treated *npq1 A. thaliana* leaves (black circles) and the *npq1* δ component (red crosses); b) NPQ induction curves for 5 mM DTT and 50 µM nigericin treated *npq1 A. thaliana* leaves (orange diamonds) and the *npq1* δ component (red crosses). The shaded area shows the associated standard error.

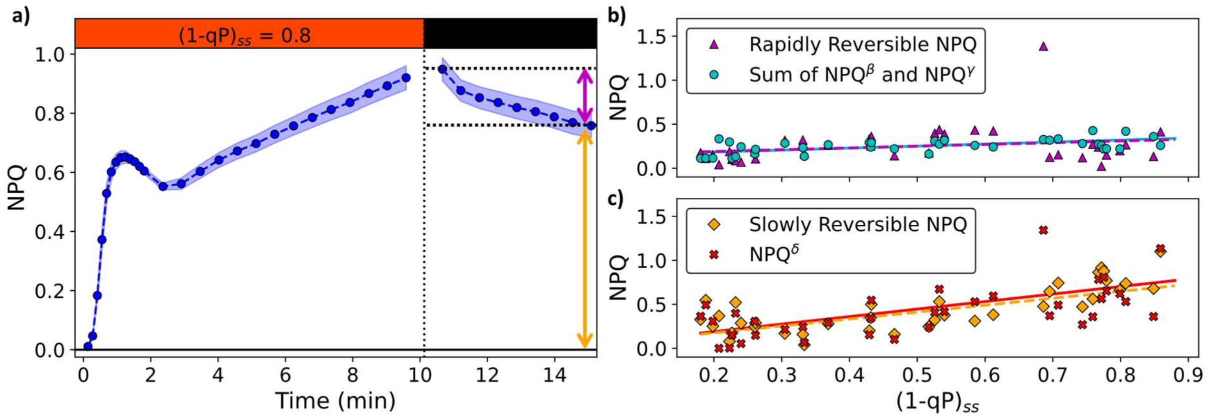

Figure S19: Non-photochemical quenching (NPQ) data illustrating the rapidly and slowly reversible NPQ seen in *npq1 A. thaliana* alongside a direct comparison between the size of the rapidly reversible NPQ and the sum of β & γ components as well as the size of the slowly reversible NPQ and the δ component. a) An average NPQ induction and post-illumination recovery curves for *npq1 A. thaliana* (blue circles) obtained at $(1\text{-qP})_{ss} = 0.8$, with double sided arrows indicating the size of the rapidly (magenta) and slowly reversible (orange) NPQ; b) a comparison between the size of the rapidly reversible NPQ (purple triangles) and the sum of the NPQ^{β} and NPQ^{γ} components (cyan circles) at the end of the illumination time. c) A comparison between the size of the slowly reversible NPQ (orange diamonds) and the NPQ^{δ} component (red crosses) at the end of the illumination time. The shaded area shows the associated standard error.

References:

Bader, A.N. *et al.* (2014) 'Phasor approaches simplify the analysis of tryptophan fluorescence data in protein denaturation studies', *Methods and Applications in Fluorescence*, 2(4), p. 045001. Available at: https://doi.org/10.1088/0000-0000/2/4/045001.

Berry, M.W. *et al.* (2007) 'Algorithms and applications for approximate nonnegative matrix factorization', *Computational Statistics and Data Analysis*, 52(1), pp. 155–173. Available at: https://doi.org/10.1016/j.csda.2006.11.006.

Franssen, W.M.J. *et al.* (2020) 'Full-Harmonics Phasor Analysis: Unravelling Multiexponential Trends in Magnetic Resonance Imaging Data', *The Journal of Physical Chemistry Letters*, 11(21), pp. 9152–9158. Available at: https://doi.org/10.1021/acs.jpclett.0c02319.

Naik, G.R. (2008) *Non-Negative Matrix Factorization Techniques and Optimizations*.

Pârvu, O. and Gilbert, D. (2016) 'Implementation of linear minimum area enclosing triangle algorithm: Application note', *Computational and Applied Mathematics*, 35(2), pp. 423–438. Available at: https://doi.org/10.1007/s40314-014-0198-8.

Torrado, B., Malacrida, L. and Ranjit, S. (2022) 'Linear Combination Properties of the Phasor Space in Fluorescence Imaging', *Sensors*, 22(3). Available at: https://doi.org/10.3390/s22030999.
